# An updated review of the pharmacological effects and potential mechanisms of hederagenin and its derivatives

**DOI:** 10.3389/fphar.2024.1374264

**Published:** 2024-06-19

**Authors:** Huize Zhang, Yong Li, Yi Liu

**Affiliations:** ^1^ School of Basic Medicine, Chengdu University of Traditional Chinese Medicine, Chengdu, China; ^2^ College of Pharmacy, Chengdu University of Traditional Chinese Medicine, Chengdu, China

**Keywords:** hederagenin, derivative, anti-cancer, anti-inflammation, pharmacology

## Abstract

Hederagenin (HG) is a natural pentacyclic triterpenoid that can be isolated from various medicinal herbs. By modifying the structure of HG, multiple derivatives with superior biological activities and safety profiles have been designed and synthesized. Accumulating evidence has demonstrated that HG and its derivatives display multiple pharmacological activities against cancers, inflammatory diseases, infectious diseases, metabolic diseases, fibrotic diseases, cerebrovascular and neurodegenerative diseases, and depression. Previous studies have confirmed that HG and its derivatives combat cancer by exerting cytotoxicity, inhibiting proliferation, inducing apoptosis, modulating autophagy, and reversing chemotherapy resistance in cancer cells, and the action targets involved mainly include STAT3, Aurora B, KIF7, PI3K/AKT, NF-κB, Nrf2/ARE, Drp1, and P-gp. In addition, HG and its derivatives antagonize inflammation through inhibiting the production and release of pro-inflammatory cytokines and inflammatory mediators by regulating inflammation-related pathways and targets, such as NF-κB, MAPK, JAK2/STAT3, Keap1-Nrf2/HO-1, and LncRNA A33/Axin2/β-catenin. Moreover, anti-pathogen, anti-metabolic disorder, anti-fibrosis, neuroprotection, and anti-depression mechanisms of HG and its derivatives have been partially elucidated. The diverse pharmacological properties of HG and its derivatives hold significant implications for future research and development of new drugs derived from HG, which can lead to improved effectiveness and safety profiles.

## 1 Introduction

Pharmacological properties and structural modifications of natural products have always inspired the exploration of new drugs. Hederagenin (HG), (3β, 4α)-3, 23-dihydroxyolean-12-en-28-oic acid, is a natural oleanane-type saponin with a molecular weight of 472.71 g/mol. The molecular formula of HG is C_30_H_48_O_4_. HG is a white and odorless crystalline powder with a bitter taste, highly insoluble in water and slightly soluble in methanol and ethanol (Zeng et al., 2018). As a target molecule of great interest, HG has attracted increasing attention.

HG is a readily available monomer compound widely distributed in a diversity of medicinal herbs. HG was initially discovered in *Hedera helix* L., also known as English ivy, a species of flowering plant belonging to the Araliaceae family, which is indigenous to a significant portion of Europe and western Asia. The designation of HG was based on its identification from this source (Xie W. et al., 2023). HG has subsequently been identified as the active component in numerous plants, originating from various families. These plants mainly belong to families of Araliaceae such as *Acanthopanax giraldii* Harms (Zhong et al., 2010), Ranunculaceae such as *Clematis chinensis* Osbeck (Li Q. Q. et al., 2013c), Rubiaceae such as *Gardenia jasminoides* Ellis. (Zhang et al., 2013), Sapindaceae such as *Sapindus mukorossi* Gaertn (Takagi et al., 1980), Caprifoliaceae such as *Lonicera japonica* Thunb. (Zhang et al., 2008), Lamiaceae such as *Perilla frutescens* (L.) Britt. (Song et al., 2014), and Lardizabalaceae such as *Fructus Akebiae* (Jin et al., 2012). In particular, the plants of Araliaceae and Ranunculaceae families have a relatively high content of HG. Notably, the seeds of *Nigella glandulifera* Freyn (a medicinal plant in Uighur medicine used to promote urination, activate blood, and remove toxin) (Shi et al., 2012) show the highest HG content. Table 1 lists the main plant species that contain HG as an active constituent.

**TABLE 1:** Main HG-containing plant species.

Plant	Family	Medicinal parts	Content (%)	References
*Beaumontia grandiflora* Wall.	Apocynaceae	Branches and leaves	0.0024	Wang et al. (2009)
*Cussonia arborea* Hochst. ex A. Rich	Araliaceae	Root-bark	Not measured	Oladimeji et al. (2017)
*Hedera helix* L.	Araliaceae	Leaves	Not measured	Liu B. X. et al. (2014a)
*Kalopanax septemlobus* (Thunb.) Koidz.	Araliaceae	Barks	1.314–1.399	Shu et al. (2019)
*Schefflera arboricola* Hayata	Araliaceae	Stems	0.07–0.31	Lu et al. (2012)
*Hedera sinensis* (Tobler) Hand.-Mazz.	Araliaceae	Whole plant	0.13–1.18	Li et al. (2020)
*Acanthopanax giraldii* Harms	Araliaceae	Leaves	0.296–2.715	Zhong et al. (2010)
*Caulophyllum robustum* Maxim.	Berberidaceae	Roots and rhizomes	0.000057	Wang J. et al. (2018a)
*Campsis grandiflora* (Thunb.) K. Schum	Bignoniaceae	Leaves	0.00039	Jin et al. (2004)
*Lonicera syringantha* Maxim.	Caprifoliaceae	Aerial parts	0.000027	Qian et al. (2006)
*Lonicera japonica* Thunb.	Caprifoliaceae	Flowers	0.35–0.81	Zhang et al. (2008)
*Gypsophila oldhamiana*	Caryophyllaceae	Roots	0.000067	Luo et al. (2008)
*Luffa cylindrica* (L.) Roem	Cucurbitaceae	Roots	0.0059–0.083	Li et al. (2013a)
*Dipsacus asper* Wall. ex Henry	Dipsacaceae	Roots	0.534–4.526	Li et al. (2013b)
*Quercus pannosa* Hand.-Mazz.	Fagaceae	Branches and leaves	0.000072	Ou et al. (2013)
*Liquidambar formosana* Hance	Hamamelidaceae	Dried resins	0.001	Xu et al. (2023)
*Cyclocarya paliurus* (Batalin) Iljinsk.	Juglandaceae	Leaves	0.0002	Yan et al. (2021)
*Galeopsis bifida* Boenn.	Lamiaceae	Whole plant	0.0013	Zhang et al. (2002)
*Perilla frutescens* (L.) Britt.	Lamiaceae	Stems	0.002	Song et al. (2014)
*Fructus Akebiae*	Lardizabalaceae	Fruits	0.0045	Jin et al. (2012)
*Akebia quinata* Decaisne	Lardizabalaceae	Stems	0.00011	Chowdhury et al. (2017)
*Viscum ovalifolium* DC.	Loranthaceae	Whole plant	0.0018	Yang Y. J. et al. (2011b)
*Syzygium grijsii* (Hance) Merr. et Perry	Myrtaceae	Stems	0.00009	Luo D. Z. et al. (2020a)
*Paeonia delavayi* Franch.	Paeoniaceae	Roots	0.00064	Wu et al. (2005)
*Pittosporum brevicalyx* (Oliv.) Gagnep.	Pittosporaceae	Barks	0.001	Yang et al. (1986)
*Nigella glandulifera* Freyn	Ranunculaceae	Seeds	3.62	Shi et al. (2012)
*Clematis chinensis* Osbeck	Ranunculaceae	Roots and rhizomes	0.09–0.66	Li et al. (2013c)
*Pulsatilla dahurica* (Fisch.) Spreng	Ranunculaceae	Roots	0.479–2.267	Zheng et al. (2012)
*Paeonia mairei* Levl.	Ranunculaceae	Roots	0.014	Shi et al. (2014)
*Clematis apiifolia* DC.	Ranunculaceae	Whole plant	0.0012	Zhou et al. (2019)
*Sarcocephalus pobeguinii* (Hua ex Pobég)	Rubiaceae	Leaves, fruits, and barks	0.00077	Mfotie Njoya et al. (2023)
*Crossopteryx febrifuga* Benth	Rubiaceae	Stem-bark	0.00023	Kayangar et al. (2019)
*Nauclea officinalis* (Pierre ex Pit.) Merr. et Chun	Rubiaceae	Branches and leaves	0.0005	Ma et al. (2005)
*Gardenia jasminoides* Ellis.	Rubiaceae	Fruits	0.000024	Zhang et al. (2013)
*Sapindus mukorossi* Gaertn	Sapindaceae	Fruit husks	Not measured	Takagi et al. (1980)
*Nephelium lappaceum* L.	Sapindaceae	Hulls	0.00022	Zhao et al. (2011)
*Sapindus saponaria* L.	Sapindaceae	Pericarp	0.425	Rodríguez-Hernández et al. (2015)
*Saururus chinensis* (Lour.) Baill.	Saururaceae	Aerial parts	0.00023	Ge et al. (2023)
*Euscaphis japonica* (Thunb.) Kanitz	Staphyleaceae	Branches	0.000071	Zhang et al. (2012)
*Aquilaria sinensis* (Lour.) Gilg	Thymelaeaceae	Wood containing resins	0.00027	Liu et al. (2007)
*Trapa acornis* Nakano	Trapaceae	Nutshells	0.0004	Chen et al. (2012)
*Boehmeria nivea* (L.) Gaudich.	Urticaceae	Roots	Not measured	Xu et al. (2009)

**FIGURE 1:**
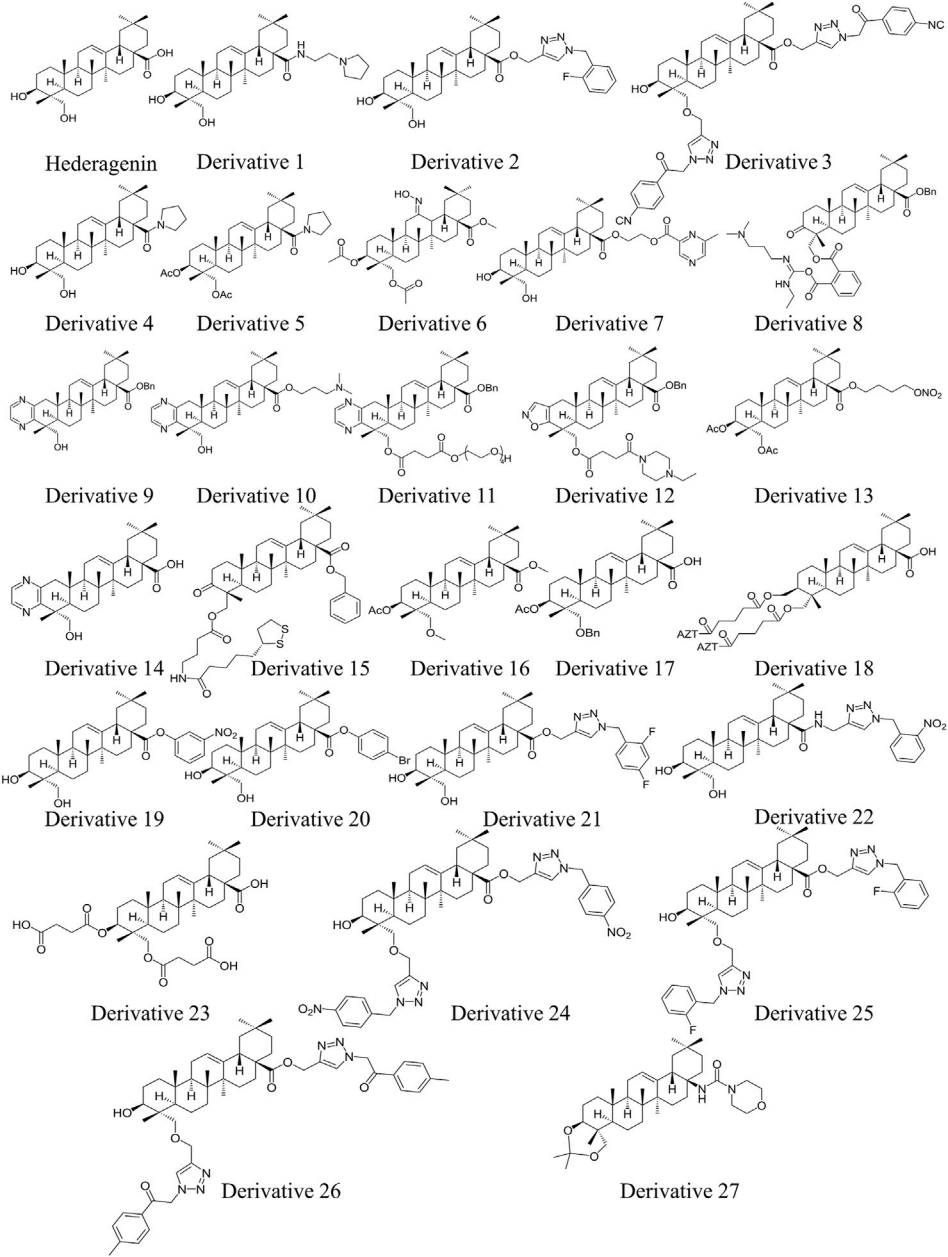
Chemical structures of HG and its derivatives 1–27 (drawn by using ChemDraw).

In terms of structure, naturally existing HG is a member of the pentacyclic triterpenoid family, consisting of a 30-carbon skeleton. HG bears a hydroxyl group at the C-3 and C-23 positions in ring A, a double bond at the C-12 and C-13 positions in ring C, and a carboxylate group at the C-28 position in ring E (Figure 1). The structural characteristics of HG suggest that it has multiple sites for structural modification, which provide a broad space for chemical transformations (Wang X. et al., 2019). Various derivatives of HG with superior biological activities and safety profiles have been synthesized through introducing different substitution groups at available sites, particularly in terms of its anti-cancer, anti-inflammation, and anti-pathogen effects. The compound names, molecular formulas, and molecular weights of the HG derivatives highlighted in the review are summarized in Table 2, and their chemical structures are depicted in Figure 1. In addition, Table 3 lists some substituents that have been shown to exhibit biological activities. For instance, introduction of ethylpyrrolidinyl group (Rodríguez-Hernández et al., 2015), triazolyl group (Rodríguez-Hernández et al., 2016b), pyrrolidinyl group (Rodríguez-Hernández et al., 2019), and pyrazine group (Fang et al., 2018) at C-28 can improve the cytotoxic activity of HG against cancer cells.

**TABLE 2:** The compound names, molecular formulas, and molecular weights of the HG derivatives presented in the review (generated using ChemDraw).

Compound	Compound name	Molecular formula	Molecular weight (g/mol)	References
Derivative 1	(4a*S*,6a*S*,6b*R*,8a*R*,9*R*,10*S*,12a*R*,12b*R*,14b*S*)-10-hydroxy-9-(hydroxymethyl)-2,2,6a,6b,9,12a-hexamethyl-*N*-(2-(pyrrolidin-1-yl)ethyl)-1,3,4,5,6,6a,6b,7,8,8a,9,10,11,12,12a,12b,13,14b-octadecahydropicene-4a(2*H*)-carboxamide	C_36_H_60_N_2_O_3_	568.89	Rodríguez-Hernández et al. (2015)
Derivative 2	(1-(2-fluorobenzyl)-1*H*-1,2,3-triazol-4-yl)methyl (4a*S*,6a*S*,6b*R*,8a*R*,9*R*,10*S*,12a*R*,12b*R*,14b*S*)-10-hydroxy-9-(hydroxymethyl)-2,2,6a,6b,9,12a-hexamethyl-1,3,4,5,6,6a,6b,7,8,8a,9,10,11,12,12a,12b,13,14b-octadecahydropicene-4a(2*H*)-carboxylate	C_40_H_56_FN_3_O_4_	661.90	Rodríguez-Hernández et al. (2016b)
Derivative 3	(1-(2-(4-isocyanophenyl)-2-oxoethyl)-1*H*-1,2,3-triazol-4-yl)methyl (4a*S*,6a*S*,6b*R*,8a*R*,9*R*,10*S*,12a*R*,12b*R*,14b*S*)-10-hydroxy-9-(((1-(2-(4-isocyanophenyl)-2-oxoethyl)-1*H*-1,2,3-triazol-4-yl)methoxy)methyl)-2,2,6a,6b,9,12a-hexamethyl-1,3,4,5,6,6a,6b,7,8,8a,9,10,11,12,12a,12b,13,14b-octadecahydropicene-4a(2*H*)-carboxylate	C_54_H_64_N_8_O_6_	921.16	Rodríguez-Hernández et al. (2017)
Derivative 4	((4a*S*,6a*S*,6b*R*,8a*R*,9*R*,10*S*,12a*R*,12b*R*,14b*S*)-10-hydroxy-9-(hydroxymethyl)-2,2,6a,6b,9,12a-hexamethyl-1,3,4,5,6,6a,6b,7,8,8a,9,10,11,12,12a,12b,13,14b-octadecahydropicen-4a(2*H*)-yl)(pyrrolidin-1-yl)methanone	C_34_H_55_NO_3_	525.82	Rodríguez-Hernández et al. (2019)
Derivative 5	((3*S*,4*R*,4a*R*,6a*R*,6b*S*,8a*S*,12a*S*,14a*R*,14b*R*)-3-acetoxy-4,6a,6b,11,11,14b-hexamethyl-8a-(pyrrolidine-1-carbonyl)-1,2,3,4,4a,5,6,6a,6b,7,8,8a,9,10,11,12,12a,14,14a,14b-icosahydropicen-4-yl)methyl acetate	C_38_H_59_NO_5_	609.89	Rodríguez-Hernández et al. (2019)
Derivative 6	methyl (4a*S*,6a*R*,6b*R*,8a*R*,9*R*,10*S*,12a*R*,12b*R*,14b*S*,*E*)-10-acetoxy-9-(acetoxymethyl)-14-(hydroxyimino)-2,2,6a,6b,9,12a-hexamethylicosahydropicene-4a(2*H*)-carboxylate	C_35_H_55_NO_7_	601.83	Liu et al. (2020a)
Derivative 7	2-(((4a*S*,6a*S*,6b*R*,8a*R*,9*R*,10*S*,12a*R*,12b*R*,14b*S*)-10-hydroxy-9-(hydroxymethyl)-2,2,6a,6b,9,12a-hexamethyl-1,2,3,4,4a,5,6,6a,6b,7,8,8a,9,10,11,12,12a,12b,13,14b-icosahydropicene-4a-carbonyl)oxy)ethyl 6-methylpyrazine-2-carboxylate	C_38_H_56_N_2_O_6_	636.87	Fang et al. (2018)
Derivative 8	2-((((4*R*,4a*R*,6a*R*,6b*S*,8a*S*,12a*S*,14a*R*,14b*R*)-8a-((benzyloxy)carbonyl)-4,6a,6b,11,11,14b-hexamethyl-3-oxo-1,2,3,4,4a,5,6,6a,6b,7,8,8a,9,10,11,12,12a,14,14a,14b-icosahydropicen-4-yl)methoxy)carbonyl)benzoic (*E*)-*N*'-(3-(dimethylamino)propyl)-*N*-ethylcarbamimidic anhydride	C_53_H_73_N_3_O_7_	864.18	Liu et al. (2017)
Derivative 9	benzyl (4a*S*,6a*S*,6b*R*,8a*R*,9*S*,14a*R*,14b*R*,16b*S*)-9-(hydroxymethyl)-2,2,6a,6b,9,14a-hexamethyl-1,3,4,5,6,6a,6b,7,8,8a,9,14,14a,14b,15,16b-hexadecahydrochryseno[1,2-*g*]quinoxaline-4a(2*H*)-carboxylate	C_39_H_52_N_2_O_3_	596.86	Yang et al. (2018)
Derivative 10	3-(dimethylamino)propyl (4a*S*,6a*S*,6b*R*,8a*R*,9*S*,14a*R*,14b*R*,16b*S*)-9-(hydroxymethyl)-2,2,6a,6b,9,14a-hexamethyl-1,3,4,5,6,6a,6b,7,8,8a,9,14,14a,14b,15,16b-hexadecahydrochryseno[1,2-*g*]quinoxaline-4a(2*H*)-carboxylate	C_37_H_57_N_3_O_3_	591.88	Wang et al. (2019a)
Derivative 11	((4a*S*,6a*S*,6b*R*,8a*R*,9*S*,14a*R*,14b*R*,16b*S*)-4a-((benzyloxy)carbonyl)-2,2,6a,6b,9,14a-hexamethyl-1,2,3,4,4a,5,6,6a,6b,7,8,8a,9,14,14a,14b,15,16b-octadecahydrochryseno[1,2-*g*]quinoxalin-9-yl)methyl (2-(2-(2-(2-hydroxyethoxy)ethoxy)ethoxy)ethyl) succinate	C_51_H_72_N_2_O_10_	873.14	Wang et al. (2021)
Derivative 12	benzyl (4a*S*,6a*S*,6b*R*,8a*R*,9*R*,13a*R*,13b*R*,15b*S*)-9-(((4-(4-ethylpiperazin-1-yl)-4-oxobutanoyl)oxy)methyl)-2,2,6a,6b,9,13a-hexamethyl-1,3,4,5,6,6a,6b,7,8,8a,9,13,13a,13b,14,15b-hexadecahydropiceno[2,3-*d*]isoxazole-4a(2*H*)-carboxylate	C_48_H_67_N_3_O_6_	782.08	Huang W. et al. (2022b)
Derivative 13	4-(nitrooxy)butyl (4a*S*,6a*S*,6b*R*,8a*R*,9*R*,10*S*,12a*R*,12b*R*,14b*S*)-10-acetoxy-9-(acetoxymethyl)-2,2,6a,6b,9,12a-hexamethyl-1,3,4,5,6,6a,6b,7,8,8a,9,10,11,12,12a,12b,13,14b-octadecahydropicene-4a(2*H*)-carboxylate	C_38_H_59_NO_9_	673.89	Chen et al. (2019)
Derivative 14	(4a*S*,6a*S*,6b*R*,8a*R*,9*S*,14a*R*,14b*R*,16b*S*)-9-(hydroxymethyl)-2,2,6a,6b,9,14a-hexamethyl-1,3,4,5,6,6a,6b,7,8,8a,9,14,14a,14b,15,16b-hexadecahydrochryseno[1,2-*g*]quinoxaline-4a(2*H*)-carboxylic acid	C_32_H_46_N_2_O_3_	506.73	Yu et al. (2023)
Derivative 15	benzyl (4a*S*,6a*S*,6b*R*,8a*R*,9*R*,12a*R*,12b*R*,14b*S*)-9-(((4-(5-((*R*)-1,2-dithiolan-3-yl)pentanamido)butanoyl)oxy)methyl)-2,2,6a,6b,9,12a-hexamethyl-10-oxo-1,3,4,5,6,6a,6b,7,8,8a,9,10,11,12,12a,12b,13,14b-octadecahydropicene-4a(2*H*)-carboxylate	C_49_H_71_NO_6_S_2_	834.23	Li et al. (2024a)
Derivative 16	methyl (4a*S*,6a*S*,6b*R*,8a*R*,9*R*,10*S*,12a*R*,12b*R*,14b*S*)-10-acetoxy-9-(methoxymethyl)-2,2,6a,6b,9,12a-hexamethyl-1,3,4,5,6,6a,6b,7,8,8a,9,10,11,12,12a,12b,13,14b-octadecahydropicene-4a(2*H*)-carboxylate	C_34_H_54_O_5_	542.80	Hong et al. (2017)
Derivative 17	(4a*S*,6a*S*,6b*R*,8a*R*,9*R*,10*S*,12a*R*,12b*R*,14b*S*)-10-acetoxy-9-((benzyloxy)methyl)-2,2,6a,6b,9,12a-hexamethyl-1,3,4,5,6,6a,6b,7,8,8a,9,10,11,12,12a,12b,13,14b-octadecahydropicene-4a(2*H*)-carboxylic acid	C_39_H_56_O_5_	604.87	Hong et al. (2017)
Derivative 18	Unable to name the compound due to the unclear binding site between hemiester and zidovudine (AZT)	-	-	Hao et al. (2021)
Derivative 19	3-nitrophenyl (4a*S*,6a*S*,6b*R*,8a*R*,9*R*,10*S*,12a*R*,12b*R*,14b*S*)-10-hydroxy-9-(hydroxymethyl)-2,2,6a,6b,9,12a-hexamethyl-1,3,4,5,6,6a,6b,7,8,8a,9,10,11,12,12a,12b,13,14b-octadecahydropicene-4a(2*H*)-carboxylate	C_36_H_51_NO_6_	593.81	Rodríguez-Hernández et al. (2016a)
Derivative 20	4-bromophenyl (4a*S*,6a*S*,6b*R*,8a*R*,9*R*,10*S*,12a*R*,12b*R*,14b*S*)-10-hydroxy-9-(hydroxymethyl)-2,2,6a,6b,9,12a-hexamethyl-1,3,4,5,6,6a,6b,7,8,8a,9,10,11,12,12a,12b,13,14b-octadecahydropicene-4a(2*H*)-carboxylate	C_36_H_51_BrO_4_	627.70	Rodríguez-Hernández et al. (2016a)
Derivative 21	(1-(2,4-difluorobenzyl)-1*H*-1,2,3-triazol-4-yl)methyl (4a*S*,6a*S*,6b*R*,8a*R*,9*R*,10*S*,12a*R*,12b*R*,14b*S*)-10-hydroxy-9-(hydroxymethyl)-2,2,6a,6b,9,12a-hexamethyl-1,3,4,5,6,6a,6b,7,8,8a,9,10,11,12,12a,12b,13,14b-octadecahydropicene-4a(2*H*)-carboxylate	C_40_H_55_F_2_N_3_O_4_	679.89	Rodríguez-Hernández et al. (2016a)
Derivative 22	(4a*S*,6a*S*,6b*R*,8a*R*,9*R*,10*S*,12a*R*,12b*R*,14b*S*)-10-hydroxy-9-(hydroxymethyl)-2,2,6a,6b,9,12a-hexamethyl-*N*-((1-(2-nitrobenzyl)-1*H*-1,2,3-triazol-4-yl)methyl)-1,3,4,5,6,6a,6b,7,8,8a,9,10,11,12,12a,12b,13,14b-octadecahydropicene-4a(2*H*)-carboxamide	C_40_H_57_N_5_O_5_	687.93	Rodríguez-Hernández et al. (2016a)
Derivative 23	(4a*S*,6a*S*,6b*R*,8a*R*,9*R*,10*S*,12a*R*,12b*R*,14b*S*)-10-((3-carboxypropanoyl)oxy)-9-(((3-carboxypropanoyl)oxy)methyl)-2,2,6a,6b,9,12a-hexamethyl-1,3,4,5,6,6a,6b,7,8,8a,9,10,11,12,12a,12b,13,14b-octadecahydropicene-4a(2*H*)-carboxylic acid	C_38_H_56_O_10_	672.86	Anderson et al. (2020)
Derivative 24	(1-(4-nitrobenzyl)-1*H*-1,2,3-triazol-4-yl)methyl (4a*S*,6a*S*,6b*R*,8a*R*,9*R*,10*S*,12a*R*,12b*R*,14b*S*)-10-hydroxy-2,2,6a,6b,9,12a-hexamethyl-9-(((1-(4-nitrobenzyl)-1*H*-1,2,3-triazol-4-yl)methoxy)methyl)-1,3,4,5,6,6a,6b,7,8,8a,9,10,11,12,12a,12b,13,14b-octadecahydropicene-4a(2*H*)-carboxylate	C_50_H_64_N_8_O_8_	905.11	Rodríguez-Hernández et al. (2017)
Derivative 25	(1-(2-fluorobenzyl)-1*H*-1,2,3-triazol-4-yl)methyl (4a*S*,6a*S*,6b*R*,8a*R*,9*R*,10*S*,12a*R*,12b*R*,14b*S*)-9-(((1-(2-fluorobenzyl)-1*H*-1,2,3-triazol-4-yl)methoxy)methyl)-10-hydroxy-2,2,6a,6b,9,12a-hexamethyl-1,3,4,5,6,6a,6b,7,8,8a,9,10,11,12,12a,12b,13,14b-octadecahydropicene-4a(2*H*)-carboxylate	C_50_H_64_F_2_N_6_O_4_	851.10	Rodríguez-Hernández et al. (2017)
Derivative 26	(1-(2-oxo-2-(*p*-tolyl)ethyl)-1*H*-1,2,3-triazol-4-yl)methyl (4a*S*,6a*S*,6b*R*,8a*R*,9*R*,10*S*,12a*R*,12b*R*,14b*S*)-10-hydroxy-2,2,6a,6b,9,12a-hexamethyl-9-(((1-(2-oxo-2-(p-tolyl)ethyl)-1*H*-1,2,3-triazol-4-yl)methoxy)methyl)-1,3,4,5,6,6a,6b,7,8,8a,9,10,11,12,12a,12b,13,14b-octadecahydropicene-4a(2*H*)-carboxylate	C_54_H_70_N_6_O_6_	899.19	Rodríguez-Hernández et al. (2017)
Derivative 27	*N*-((4a*R*,4b*R*,6a*R*,6b*S*,8a*S*,12a*S*,14a*R*,14b*R*,16a*S*)-2,2,4a,6a,6b,11,11,14b-octamethyl-4a,5,6,6a,6b,7,8,9,10,11,12,12a,14,14a,14b,15,16,16a-octadecahydro-4*H*-piceno[3,4-*d*][1,3]dioxin-8a(4b*H*)-yl)morpholine-4-carboxamide	C_37_H_60_N_2_O_4_	596.90	Chakroborty et al. (2023)

**TABLE 3:** Some substituents with biological activities.

Substituents	Structural modification sites	Biological activities	References
Ethylpyrrolidinyl	C-28	Improving cytotoxic activity against cancer cells	Rodríguez-Hernández et al. (2015)
Triazolyl	C-28	Improving cytotoxic activity against cancer cells	Rodríguez-Hernández et al. (2016b)
Triazolyl	C-23 and C-28	Improving cytotoxic activity against cancer cells	Rodríguez-Hernández et al. (2017)
Pyrrolidinyl	C-28	Improving cytotoxic activity against cancer cells	Rodríguez-Hernández et al. (2019)
Acetyl	C-3 and C-23	Improving cytotoxic activity against cancer cells	Rodríguez-Hernández et al. (2019)
Oxime	C-12	Improving cytotoxic activity against cancer cells	Liu X. et al. (2020a)
Pyrazine	C-28	Improving cytotoxic activity against cancer cells	Fang et al. (2018)
Polyamine	C-23	Improving anti-proliferative activity	Liu et al. (2017)
Benzyl	C-28	Exerting chemotherapy resistance reversal activity	Yang et al. (2018)
Pyrazine	Ring A	Exerting chemotherapy resistance reversal activity	Yang et al. (2018)
3-Dimethylamino-1-isopropanol	C-28	Improving aqueous solubility	Wang X. et al. (2019a)
Polyethylene glycol	C-23 or C-28	Improving aqueous solubility	Wang et al. (2021)
Isoxazole	Ring A	Exerting chemotherapy resistance reversal activity	Huang W. et al. (2022b)
Ethyl piperazine	C-23	Exerting chemotherapy resistance reversal activity	Huang et al. (2022b)
Pyrazine	Ring A	Improving anti-inflammatory activity	Yu et al. (2023)
Benzyl	C-28	Improving anti-inflammatory activity	Li H. et al. (2024a)
Disulfide	C-23	Improving anti-inflammatory activity	Li et al. (2024a)
Triazolyl	C-23 and C-28	Improving anti-leishmanial activity	Rodríguez-Hernández et al. (2017)
Disuccinate	C-3 and C-23	Improving selectivity against *Leishmania*	Anderson et al. (2020)
Morpholine ring	C-28	Improving anthelmintic activity and selectivity against liver flukes	Chakroborty et al. (2023)

HG possessing exceptional potential for practical use, displays broad application prospects. As evidenced by numerous research articles, HG and its derivatives exhibit a wide spectrum of pharmacological activities *in vitro* and *in vivo*, such as anti-cancer (Liu B. X. et al., 2014), anti-inflammation (Lee et al., 2015), anti-neurodegeneration (Wu et al., 2017), anti-depression (Zhou et al., 2010), anti-hyperglycemia (Luo et al., 2008), anti-hyperlipidemia (Lu et al., 2015), and anti-pathogen activities (Rodríguez-Hernández et al., 2016a). Recently, the novel pharmacological effects are constantly being discovered, such as anti-fibrosis effect (Ma et al., 2020; Yang and He, 2022) and anti-osteoporosis effect (Tian et al., 2020; Huai et al., 2021). In addition, more and more neoteric target genes and signaling pathways through which HG and its derivatives exert their effects have been experimentally validated. Some researchers have concentrated on the recent advances of HG and its derivatives (Zeng et al., 2018; Xie W. et al., 2023a; Huang et al., 2023). However, an action target-focused review concerning recent pharmacological studies in this field is missing. Therefore, we teased out the action targets of HG and its derivatives that exert various pharmacological effects from the up-to-date and some classical research, and discussed their pharmacokinetic characteristics, safety, targeting, and bioavailability.

In this study, information from the PubMed, Web of Science, and Chinese National Knowledge Infrastructure was obtained using the search term “Hederagenin”. The data collected for this updated review is current to February 2024. The inclusion criteria were that the studies scrutinized the pharmacological effects and underpinning mechanisms of pure HG or its derivatives, while research that did not employ HG or its derivatives as the single active component was excluded. Through a thorough examination of the included literature, this review aims to offer a useful reference for future investigations and accelerate the clinical application of HG and its derivatives.

## 2 Effects and mechanisms of HG and its derivatives

### 2.1 Cancers

**FIGURE 2:**
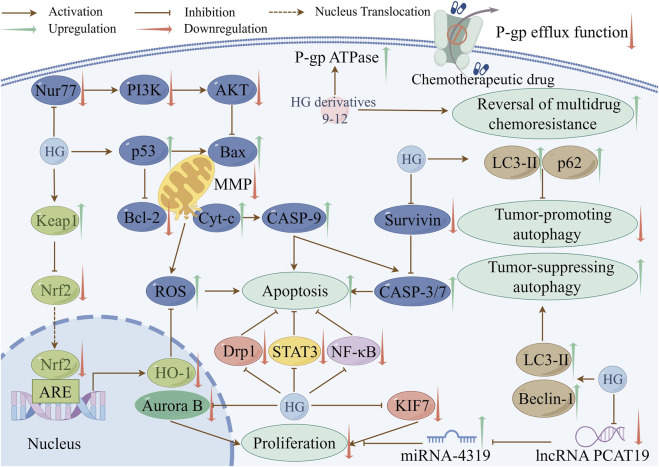
Some pathways involved in the anti-cancer mechanisms of HG and its derivatives (created with www.figdraw.com).

The processes of cancer cell proliferation, apoptosis, and autophagy are closely interconnected. For example, apoptosis contributes to the inhibition of cancer cell proliferation (Wong, 2011), and autophagy can impede tumor cell proliferation and stimulate tumor cell apoptosis during tumorigenesis (Ma et al., 2023; Wen and Klionsky, 2020). Their interference can promote tumor growth and survival. Therefore, it is critical to devise therapeutic strategies that target these three mechanisms in the treatment of cancers. Numerous studies have shown that HG and its derivatives have good anti-cancer properties. In addition to inhibiting proliferation, inducing apoptosis, regulating autophagy, the mechanisms also involve exerting cytotoxic activity and reversing chemotherapy resistance in cancer cells. Table 4 summarizes the anti-cancer mechanisms of HG and its derivatives, and some of the molecular pathways involved are shown in Figure 2.

**TABLE 4:** The mechanisms underlying the anti-cancer effect of HG and its derivatives.

Compound	Concentration	Model	Types of cancers	IC_50_/EC_50_	Molecular mechanisms	References
Cytotoxic activity						
Derivative 4	6 μM	A2780 cells	Ovarian cancer	3 μM (EC_50_)	Permeable cell membrane and secondary necrosis	Rodríguez-Hernández et al. (2019)
Derivative 7	2, 5, 10 μM	A549 cells	Lung cancer	3.45 μM (IC_50_)	Induced early cell apoptosis and cell-cycle arrest in the synthesis phase	Fang et al. (2018)
Inhibition of proliferation						
HG	84.62 μM	CaSki cells	Cervical cancer	84.62 μM (IC_50_)	Inhibited the STAT3 signaling pathway	Fang et al. (2019)
HG	10, 20 μM	A549 cells	Lung cancer	Not measured	Downregulated the Aurora B pathway and inhibited the expression and activity of Aurora kinases	Baek et al. (2022)
HG	10.58, 21.16, 42.31 μM	TPC-1 cells	Thyroid cancer	Not measured	Inhibited lncRNA PCAT19 expression, promoted miRNA-4319 expression, and induced cell cycle arrest in Gap 0/Gap 1 phase	Zheng et al. (2022)
HG	5, 10, 20 μM	U251 and U87 cells	Glioma	Not measured	Decreased Nur77 expression and suppressed the PI3K/AKT signaling pathway	Dai et al. (2023)
HG	84.62 μM	U87 cells	Glioma	Not measured	Regulated the hedgehog signaling pathway by reducing the expression of KIF7	Zhang et al. (2023)
Induction of apoptosis						
HG	9.44, 18.87, 37.74 μM	MCF-7 cells	Breast cancer	18.87 μM (IC_50_)	Induced cell death via caspase-3/-7 activation	Mfotie Njoya et al. (2023)
HG	10 μM	HepG2 cells	Liver cancer	72.37 μM (IC_50_)	Decreased phosphorylated AKT expression and increased the expression of p53	Wu et al. (2019b)
HG	1, 2 μM	LoVo cells	Colon cancer	1.39 μM (24 h), 1.17 μM (48 h) (IC_50_)	Disrupted the MMP and induced the release of ROS	Liu et al. (2014a)
HG	70 mg/kg	MCF-7 tumor-bearing mice	Breast cancer	Not measured	Reduced the MMP and increased the production of ROS	Shang et al. (2019)
HG	10, 32, 100 μM	A549 cells	Lung cancer	26.3 μM (IC_50_)	Interrupted the MMP and suppressed NF-κB activation	Gao et al. (2016)
HG	10, 50, 80 μM	HNC cells	Head and neck cancer	Not measured	Downregulated the MMP, increased ROS production and GSH depletion, and inhibited the Nrf2/ARE pathway	Kim et al. (2017a)
HG	20 μM	SKOV3 and A2780 cells	Ovarian cancer	78.12 μM and 67.07 μM (IC_50_)	Suppressed dynamin-related protein 1 -regulated mitochondrial fission	Su et al. (2024)
Derivative 8	10 μM	MKN45 cells	Gastric cancer	7 μM (IC_50_)	Reduced the MMP	Liu et al. (2017)
Regulation of autophagy						
HG	70 mg/kg	MCF-7 tumor-bearing mice	Breast cancer	Not measured	Facilitated the generation of autophagosomes, improved Beclin-1, and encouraged LC3-I transformation into LC3-II	Shang et al. (2019)
HG	50 μM	NCI-H1299 and NCI-H1975 cells	Lung cancer	Not measured	Upregulated the levels of LC3-II and p62	Wang et al. (2020)
Reversal of chemotherapy resistance in cancer cells						
Derivative 9	5, 10 μM	KBV and MCF7/T cells	Oral cancer and breast cancer	Not measured	Blocked P-gp drug efflux pump function by enhancing the activity of P-gp ATPase	Yang et al. (2018)
Derivative 10	0.5, 1, 2, 5 μM	KBV cells	Oral cancer	Not measured	Inhibited the efflux function of P-gp by stimulating P-gp ATPase activity	Wang et al. (2019a)
Derivative 11	5, 10 μM	KBV and MCF-7/T cells	Oral cancer and breast cancer	Not measured	Suppressed the efflux function of P-gp	Wang et al. (2021)
Derivative 12	2.5, 5, 10 μM	KBV cells	Oral cancer	Not measured	Inhibited P-gp efflux function by activating P-gp ATPase	Huang W. et al. (2022b)
Derivative 13	100 μM	H1975 and H1975-LTC cells	Lung cancer	8.1 μM and 7.6 μM (IC_50_)	Improved the level of NO	Chen et al. (2019)
HG	10 μM	AGS cells	Gastric cancer	Not measured	Suppressed activation of the PI3K/AKT signaling pathway	Tang et al. (2024)

#### 2.1.1 Cytotoxicity

##### 2.1.1.1 Cytotoxicity of HG

The selective cytotoxicity of the candidate drug to cancer cells directly determines its anti-cancer activity. The stronger the selective cytotoxicity against tumor cells, the better the anti-cancer effect. HG is cytotoxic to various cancer cell lines. HG exhibited moderate cytotoxicity against the A549 tumor cell line, as evidenced by its half-maximal inhibitory concentration (IC_50_) value of 39 µM (Gauthier et al., 2009). HG was also found to selectively display potent cytotoxicity toward CEM cells with an IC_50_ value of 2.11 μM (Zhang et al., 2012). Additionally, Wang et al. reported that HG exerted cytotoxic effects on several tumor cell lines consisting of HL-60, A549, HeLa, HepG2, and U87MG, with IC_50_ values varying from 27.52 to 42.27 μM (Wang et al., 2013). Moreover, recent research has also indicated that HG selectively inhibited the proliferation of four human cancer cell lines with IC_50_ values in the range of 18.87–77.61 μM (MCF-7, HepG2, Caco-2, and A549) (Mfotie Njoya et al., 2023).

##### 2.1.1.2 Cytotoxicity of HG derivatives

Due to the restricted anti-cancer capability of HG, many HG derivatives with enhanced cytotoxic properties have been synthesized. Diego Rodríguez-Hernández and colleagues conducted a series of studies on the structural modifications of HG. Thirty different C-28 ester and amide derivatives of HG were synthesized, and their cytotoxic properties were evaluated in the 518A2, A2780, HT29, MCF7, A549, and 8505C cancer cell lines. Most of the novel compounds exhibited greater cytotoxicity against all cell lines compared to HG, and half-maximal effective concentration (EC_50_) values of HG varied from 19.9 to 50.0 µM. The most effective compound was derivative **1**, an amide carrying an ethylpyrrolidinyl group, whose EC_50_ values were in the range of 1.1–3.7 µM (Figure 1) (Rodríguez-Hernández et al., 2015). Moreover, the majority of another 30 new HG derivatives with 1,2,3-triazolyl attached to C-28 manifested superior anti-cancer activities against the 6 cell lines in the former study in comparison with natural HG. Notably, derivative **2** (Figure 1), an ester derivative containing an *o*-F group, selectively exhibited the most potent cytotoxic activity against human colon adenocarcinoma HT29 cells, with an EC_50_ value of 1.6 µM (Rodríguez-Hernández et al., 2016b). In addition, 18 bis-triazolyl derivatives modified with C-23 and C-28 were synthesized, and their cytotoxic effects were tested in the FaDu, HT29, A2780, A375, and SW1736 tumor cell lines. Derivative **3** (Figure 1) with a substituted 2-(*p*-cyanophenyl)-2-oxoethyl group in the triazolyl core, exhibited the highest cytotoxicity against all human cancer cell lines, with EC_50_ values in the range of 7.4–12.1 µM (Rodríguez-Hernández et al., 2017). In a recent study, a sequence of amide derivatives of HG containing nitrogen heterocycles at C-28, including or excluding an acetyl group at C-3 and C-23 in ring A, were synthesized. The study showed that a large percentage of these derivatives were cytotoxic to the MCF7, FaDu, A2780, HT29, and SW1736 tumor cell lines. Particularly, among the hydroxylated and acetylated derivatives, the most cytotoxic compounds were derivatives **4** (EC_50_ = 1.2–3.6 µM) and **5** (EC_50_ = 0.4–2.5 µM), respectively (Figure 1), both of which bear a pyrrolidinyl group at C-28. Moreover, the latter displayed higher selectivity and lower toxicity. The mechanisms underlying the cytotoxic effect of derivative **4** might be correlated with the permeability of the cell membrane and secondary necrosis (Rodríguez-Hernández et al., 2019).

In another study concerning HG derivatives by Liu et al., HG was converted into a series of new derivatives, most of which demonstrated anti-proliferative effects and superior selectivity against the HepG2 cell line. Derivative **6** (Figure 1), carrying an oxime group at C-12, displayed the most potent anti-hepatoma activity with an IC_50_ value of 1.88 μM, the highest selectivity, and the lowest toxicity. This indicates that altering the structure of the C ring on the HG backbone is crucial for regulating anti-hepatoma activity (Liu X. et al., 2020). Furthermore, 26 pyrazine derivatives of HG were designed and synthesized, and most of them were more potent than HG in terms of their cytotoxic effects on three human tumor cell lines (A549, MCF-7, and HepG2). Notably, derivative **7** coupled with 2,6-dimethylpiperazine via a two-carbon chain (Figure 1) was the most effective candidate for anti-cancer therapy. It exhibited IC_50_ values ranging from 3.45 to 8.73 μM, which could concentration-dependently induce early cell apoptosis and trigger cell-cycle arrest in the synthesis phase of A549 cells (Fang et al., 2018). These studies pertaining to modification of the skeletal structure of HG have demonstrated that the introduction of various substituent groups at available locations, including C-28, C-23, C-3, ring A, and ring C, can enhance the cytotoxicity and selectivity of HG toward tumor cells. The successful development of HG derivatives can serve as a source of inspiration for future research concerning its structural modifications.

#### 2.1.2 Inhibition of proliferation

Cancer is fundamentally characterized by unregulated cell proliferation, and the excessive multiplication of cancer cells can expedite tumor formation and development (Zhu and Thompson, 2019). One of the primary mechanisms by which HG exerts its anti-cancer effects is impeding the proliferation of cancer cells. HG has demonstrated anti-proliferative effects in a wide variety of cancer cells. The pertinent mechanisms are outlined below.

##### 2.1.2.1 STAT3

As a critical member of the signal transducer and activator of transcription (STAT) family, STAT3 participates in tumor cell proliferation, differentiation, and apoptosis. Abnormal and continuous stimulation of STAT3 can lead to tumor growth by promoting proliferation and inhibiting apoptosis (Subotički et al., 2019). Fang et al. examined the anti-cervical cancer activity of HG using CaSki cells, and the results suggested that HG inhibited the proliferation of cervical cancer cells by decreasing the level of phosphorylated STAT3 protein and inhibiting the STAT3 signaling pathway. Furthermore, this study revealed that HG increased the levels of cleaved caspase-9 and caspase-3 to promote tumor cell apoptosis (Fang et al., 2019).

##### 2.1.2.2 Aurora B

Aurora kinase B is a mitotic kinase responsible for the segregation of chromosomes and advancement of the cell cycle, and its overexpression contributes to tumorigenesis (Kanda et al., 2005). Baek et al. determined that HG significantly suppressed proliferation, presenting as a marked reduction in the cell viability and colony-forming ability of the A549 and H1299 human non-small cell lung cancer (NSCLC) cell lines. The underlying mechanism involved the downregulation of the Aurora B pathway and inhibition of the expression and activity of Aurora kinases (Baek et al., 2022).

##### 2.1.2.3 Hedgehog

The hedgehog signaling pathway regulates intricate developmental processes, and ungoverned stimulation of hedgehog signaling is responsible for the initiation and maintenance of tumors (Pak and Segal, 2016). In the most recent study of the anti-glioma effect, HG inhibited the proliferation of the U87 human glioma cell line in a concentration-dependent manner by decreasing the expression of kinesin family member 7 (KIF7), which is an important component of the hedgehog signaling pathway (Zhang et al., 2023).

##### 2.1.2.4 LncRNA and miRNA

Long noncoding RNAs (lncRNAs) and microRNAs (miRNAs) have been demonstrated to have a significant impact on tumorigenesis and the advancement of various types of cancers (Xu et al., 2020). A study conducted by Zheng et al. found that HG promoted cell cycle arrest in the Gap 0/Gap 1 phase and suppressed the proliferation, migration, and invasion of human thyroid cancer TPC-1 cells by inhibiting lncRNA PCAT19 expression and promoting miRNA-4319 expression (Zheng et al., 2022).

##### 2.1.2.5 PI3K/AKT

The phosphatidylinositol 3 kinase (PI3K)/protein kinase B (AKT) signaling pathway controls multiple cellular biological processes and is intimately associated with the proliferation, apoptosis, and migration of tumor cells (Xue et al., 2020). The nuclear receptor Nur77, which belongs to the superfamily of steroid/thyroid hormone receptors, can target PI3K/AKT activity (Shi et al., 2021). HG depressed the proliferation, migration, and invasion and promoted apoptosis of the U251 and U87 human glioma cell lines by decreasing the gene and protein expression of Nur77. Meanwhile, the PI3K/AKT signaling pathway, which is located downstream of Nur77, was suppressed (Dai et al., 2023).

The aforementioned findings indicate that HG has the potential to affect multiple genes and signaling pathways to suppress cancer proliferation. The exploration of novel mechanisms of HG holds promise for developing innovative HG-based cancer therapeutic approaches. Nevertheless, further investigations are necessary to validate these mechanisms as effective therapeutic targets for cancer treatment.

#### 2.1.3 Induction of apoptosis

Apoptosis is a programmed process of cell death that autonomously eliminates harmful and senescent cells in the body. In general, apoptosis is inhibited in tumor cells. Through triggering apoptosis, tumor progression can be halted (Wong, 2011). Several studies have confirmed that HG and its derivatives induce tumor cell apoptosis by activating the intrinsic mitochondrial pathway. The Bcl-2 family of proteins encompasses apoptosis-promoting proteins, such as Bad and Bax, and apoptosis-suppressing proteins, such as Bcl-2 and Bcl-xL (Volkmann et al., 2014). These proteins collaborate to regulate mitochondria-mediated apoptosis by decreasing the mitochondrial membrane potential (MMP) and increasing mitochondrial membrane permeability, leading to the breakdown of mitochondrial membrane integrity. The damaged mitochondria release reactive oxygen species (ROS) and proapoptotic-related factors, such as cytochrome c (cyt c), triggering initiation of the caspase cascade pathway, which is involved in the terminal phase of apoptosis (Wang et al., 2008; Susnow et al., 2009).

HG was found to induce cell death in human breast adenocarcinoma MCF-7 cells through promoting apoptosis, as shown by the upregulation of caspase-3 and caspase-7, which are two executioner caspases in the caspase cascade pathway (Mfotie Njoya et al., 2023). A study of hepatoma indicated that HG inhibited cell viability and induced apoptosis in HepG2 cells by elevating the level of cleaved caspase-3 and reducing the Bcl-xL/Bad ratio, which might be linked to the decrease in phosphorylated AKT expression and the increase in p53 expression (Wu R. et al., 2019b). A study of the anti-colon cancer effect revealed that HG could significantly suppress the proliferation of LoVo cells in a time- and dose-dependent manner. Tumor growth inhibition mediated by HG was related to the induction of apoptosis through the mitochondrial pathway, as exemplified by disrupting MMP, inducing the generation of ROS, elevating the activation of caspase-9 and caspase-3, upregulating Bax, and downregulating Bcl-2, Bcl-xL, and Survivin. Remarkably, HG did not affect the activation of caspase-8, which initiates the extrinsic apoptotic pathway mediated by the death receptor, demonstrating that mitochondria participate in HG-induced apoptosis (Liu B. X. et al., 2014a). In a study concerning breast cancer, HG reduced the MMP, increased the production of ROS, upregulated Bax, Caspase-9, and Caspase-3, downregulated Bcl-2, and stimulated cyt c release, thereby promoting cell apoptosis mediated by the mitochondrial pathway (Shang et al., 2019). The following targets are involved in the mitochondrial apoptosis induced by HG.

##### 2.1.3.1 NF-κB

The transcription factor nuclear factor-kappa B (NF-κB) acts as an anti-apoptotic factor, therefore suppressing the NF-κB pathway has been utilized as a cancer treatment strategy (Monisha et al., 2017). HG selectively exhibited superior cytotoxicity toward the A549 and BT20 cell lines, presenting IC_50_ values of 26.3 and 11.8 µM respectively. In this study, researchers also verified that HG induced the apoptosis of A549 cells in a concentration-dependent manner through interruption of the MMP, indicating that the intrinsic pathway is involved in HG-induced apoptosis and mitochondria play a significant role in this process. The induction of apoptosis by HG may involve suppression of NF-κB activation, due to inhibition of NF-κB translocation from the cytoplasm to the nucleus (Gao et al., 2016).

##### 2.1.3.2 Nrf2/ARE

Nuclear factor erythroid 2-related factor 2 (Nrf2) is crucial for regulating the cellular redox balance. Nrf2 attaches to the antioxidant response element (ARE) in the promoter areas of its target genes, thereby stimulating the transcription of antioxidant genes (Hu et al., 2019). Activation of Nrf2/ARE pathway increases the antioxidant reaction, decreases ROS levels, and facilitates tumor progression. A study on head and neck cancer (HNC) showed that HG selectively induced apoptosis in HNC cells by enhancing the levels of Bax, cleaved poly ADP-ribose polymerase (PARP), and cleaved caspase-3 and decreasing the level of Bcl-2. The molecular mechanisms underlying these effects of HG were concerned with downregulation of the MMP, increase in ROS production, and promotion of glutathione (GSH) depletion through inhibiting the Nrf2/ARE pathway (Kim E. H. et al., 2017).

##### 2.1.3.3 Drp1

Dynamin-related protein 1 (Drp1) is essential in the division of mitochondria. Drp1-mediated mitochondrial division functions as an anti-apoptotic mechanism, therefore cancer cell apoptosis can be induced by inhibiting Drp1 activity (Inoue-Yamauchi and Oda, 2012). In a study on ovarian cancer, treatment with HG inhibited the proliferation of SKOV3 and A2780 cells and triggered mitochondria-mediated apoptosis via decreasing the level of Bcl-2, increasing the levels of Bax, Bak, caspase-9 and caspase-3, and reducing the MMP. The underlying molecular mechanism of these effects involved the inhibition of mitochondrial fission regulated by Drp1 (Su et al., 2024).

The effects of HG derivatives on inducing tumor cell apoptosis have also been studied. Liu et al. synthesized 24 derivatives of HG, and among them, polyamine derivative **8** containing three nitrogen atoms at C-23 (Figure 1) minimized the viability of MKN45 and KB tumor cells, thus exhibiting the greatest anti-cancer capacity. The anti-proliferative activity of derivative **8** was related to the induction of mitochondria-mediated apoptosis, as shown by the disrupted MMP, increased cleaved PARP and Bax, and decreased expression of Bcl-2 (Liu et al., 2017).

#### 2.1.4 Regulation of autophagy

Autophagy is a degradation and recycling process of intracellular substances and exhibits both tumor-suppressing and tumor-promoting effects on tumor development (Amaravadi et al., 2019). Three processes are involved in autophagy: the formation of autophagosomes, the fusion of autophagosomes and lysosomes, and the degradation of autophagosomes (Lian et al., 2021). Light chain 3 (LC3) is commonly used as a marker for autophagosomes, and elevated levels of LC3 may be linked to either augmented synthesis of autophagosomes or diminished degradation of autophagosomes. During formation of the autophagosome, a cytosolic form of LC3 (LC3-I) undergoes conjugation with phosphatidylethanolamine to generate the LC3-phosphatidylethanolamine conjugate (LC3-II), which is localized on the membrane of the autophagosome (Maiuri et al., 2007). Beclin-1 plays a crucial role in autophagosome formation, and its upregulation indicates the activation of autophagy (Shrestha et al., 2018). Another autophagy marker, p62, is primarily degraded during autophagy, presenting a negative correlation with autophagy (Liang et al., 2018). HG possesses different regulatory effects on autophagy. In addition to triggering tumor-suppressing autophagy to combat cancer, HG can also block tumor-promoting autophagy to increase the cytotoxicity of chemotherapy.

##### 2.1.4.1 Promotion of tumor-suppressing autophagy

In theory, autophagy can degrade aberrantly folded or long-lasting proteins and damaged cellular organelles, thereby promoting tumor cell apoptosis and functioning as a tumor inhibitor (Levy et al., 2017). In a breast cancer-bearing mouse model, Shang et al. discovered that HG activated autophagy by facilitating the generation of autophagosomes, increasing the expression of Beclin-1, and encouraging the transformation of LC3-I to LC3-II, concurrently promoting mitochondria-mediated apoptosis, to exert its anti-breast cancer effect (Shang et al., 2019).

##### 2.1.4.2 Inhibition of tumor-promoting autophagy

With the progression of tumors, autophagy exhibits a tumor-promoting effect by safeguarding tumor cells against external stimulus-induced damage, concomitant with the development of chemoresistance in tumor cells (Kondo et al., 2005). Inhibition of tumor-promoting autophagy has been disclosed to boost the effectiveness of standard chemotherapy (Sui et al., 2013). In the highly metastatic human lung cancer cell lines NCI-H1299 and NCI-H1975, which were used to represent advanced lung cancer cells, HG treatment upregulated the levels of LC3-II and p62. Moreover, HG failed to enhance the LC3-II level in the presence of the lysosomal acidification inhibitor bafilomycin A1, which can impede LC3-II degradation. These results indicated that HG blocked autophagic degradation and inhibited late autophagic flux by restricting lysosomal acidification instead of stimulating autophagy initiation. The same study also observed that HG enhanced the cytotoxicity of cisplatin and paclitaxel in lung cancer cells through augmenting the level of cytotoxic ROS and inhibiting autophagy, which can prevent tumor cell death (Wang et al., 2020). In clinical practice, most patients with cancer are diagnosed at a fairly advanced stage, and the novel autophagy inhibitor HG may improve the clinical treatment of cancer.

#### 2.1.5 Reversal of chemotherapy resistance in cancer cells

Chemotherapy remains the primary therapy for most cancers (Yang et al., 2017; Li K. et al., 2019). However, the progression of chemotherapy resistance poses a significant challenge to the efficacy of chemotherapy. Therefore, agents to reverse chemotherapy resistance have been the focus of cancer therapy research. HG and its derivatives have been demonstrated to reverse chemotherapy resistance through multiple targets.

##### 2.1.5.1 P-gp

P-glycoprotein (P-gp) acts as a transmembrane pump that can facilitate the removal of anti-cancer agents by utilizing energy released from ATP hydrolysis, and P-gp overexpression plays a significant role in the development of multidrug resistance (MDR) in tumors (Shen et al., 2020). Inhibiting P-gp protein expression and blocking its efflux function can suppress P-gp-mediated MDR (Zheng et al., 2019).

A series of HG derivatives that can reverse multidrug chemoresistance of tumors by targeting P-gp have been synthesized. The novel HG derivative **9** (Figure 1), also referred to as H6, resensitized the KBV and MCF-7/T MDR cancer cell lines to paclitaxel and vincristine. Mechanistically, H6 reversed P-gp-induced MDR by blocking P-gp drug efflux pump function via an increase in P-gp ATPase activity. Cotreatment of H6 with paclitaxel was effective and relatively safe *in vivo* (Yang et al., 2018). These findings suggest that H6 can be used as a potent adjuvant therapy in conjunction with standard chemotherapies to improve the efficiency of cancer treatment and to prevent MDR. Based on H6 with poor solubility, 29 novel H6 derivatives were synthesized to further improve anti-MDR properties. Administration of derivative **10** (Figure 1), bearing a nitrogen-containing group at C-28 and a pyrazine ring fused to ring A, led to greater MDR reversal than H6 through the identical P-gp inhibition mechanism. The combination of derivative 10 and paclitaxel induced cell cycle arrest at the Gap 2/mitosis phase and apoptosis in KBV cells. *In vivo*, oral gavage of derivative 10 exhibited the same activity as intraperitoneal injection of the parent H6 due to the improved water solubility with the nitrogen-bearing group at C-28 (Wang X. et al., 2019a). Nevertheless, neither the anti-MDR activity nor the aqueous solubility of derivative 10 was satisfactory, so polyethylene glycol (PEG) molecules with robust solubilizing ability were introduced at C-23 or C-28 of H6 to produce some PEGylated derivatives. Among them, derivative **11** (Figure 1) had improved water solubility, excellent chemical stability, and the highest tumor MDR reversal activity. The anti-MDR mechanism of derivative 11 remained the inability of MDR tumor cells to exclude chemotherapeutic drugs from the body via P-gp inhibition (Wang et al., 2021). A subsequent study confirmed that incorporating nitrogen-containing heterocycles could substantially enhance the MDR reversal activity of HG derivatives via functional suppression of P-gp. Among the synthesized derivatives, derivative **12** (Figure 1) increased the sensitivity of KBV cells to chemotherapeutic drugs and exhibited a comparatively powerful ability to reverse MDR (Huang W. et al., 2022b). This derivative has a ring A fused with an isoxazole ring, and splices the ethyl piperazine group at C-23 by using succinic anhydride as a linker.

The above experiments suggest that these derivatives have great potential as P-gp inhibitors and can be used to overcome multidrug chemoresistance, which is a pressing issue in clinical oncology that requires immediate resolution. Further research will enhance the value and utility of these derivatives in both laboratory and clinical settings.

##### 2.1.5.2 PI3K/AKT

The PI3K/AKT pathway has a significant impact on the emergence of resistance to chemotherapy. Moreover, blocking the PI3K/AKT pathway has been found to effectively reverse chemoresistance in various types of tumors (Burris, 2013). A recent study on gastric cancer confirmed that HG might decrease the resistance of oxaliplatin-resistant AGS cells to oxaliplatin through suppressing activation of the PI3K/AKT signaling pathway. Moreover, HG combined with oxaliplatin inhibited the growth of gastric cancer cells more significantly than oxaliplatin alone (Tang et al., 2024).

##### 2.1.5.3 NO

Higher levels of nitric oxide (NO) inhibit cancer progression by triggering programmed cell death, enhancing the sensitivity of tumors to chemotherapy, and reversing resistance to chemotherapy (Huang et al., 2017). Combining an NO donor with an anticancer agent may provide an appealing option for cancer treatment. Acquired resistance to epidermal growth factor receptor (EGFR) tyrosine kinase inhibitors occurs in nearly all NSCLC patients harboring EGFR-activating mutations (Nilsson et al., 2020). Chen et al. synthesized several new HG-NO donor hybrids, among which derivative **13** (Figure 1) displayed better anti-cancer activity than HG. Moreover, this derivative strongly inhibited the activity of mutant EGFR-L858R/T790M/C797S kinase and the proliferation of previous generations of EGFR tyrosine kinase inhibitor-resistant H1975 and H1975-LTC NSCLC cell lines, as well as generating the most nitrite via the synergistic effect of HG and NO donors (Chen et al., 2019). This study suggests that derivative 13 can help to overcome acquired EGFR tyrosine kinase inhibitor resistance in EGFR mutant NSCLC by increasing the level of NO through its unique chemical structure.

### 2.2 Inflammatory diseases

**FIGURE 3:**
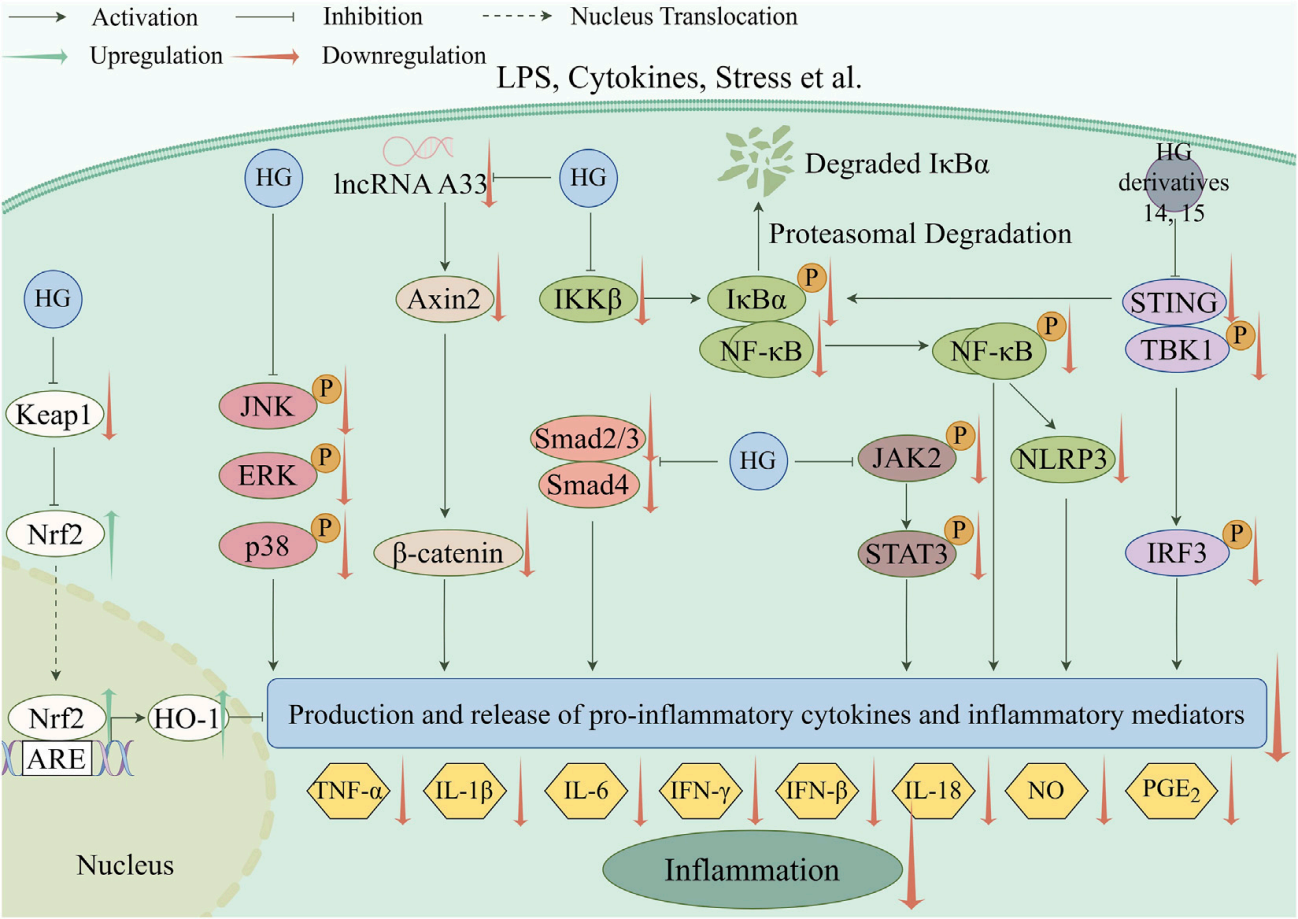
Anti-inflammatory pathways targeted by HG and its derivatives (created with www.figdraw.com).

Inflammation, which is a fundamental pathological process, contributes to the emergence of various diseases (Chen et al., 2022). The anti-inflammatory effect of HG was initially documented in two classic inflammatory animal models, including rats with hind paw edema induced by carrageenin (Takagi et al., 1980) and mice with ear edema provoked by arachidonic acid or croton oil (Kim et al., 1999). In recent years, the underlying mechanisms of the anti-inflammatory action of HG and its derivatives have been widely studied in many new animal models of inflammation. The main mechanism involves suppressing the generation and release of pro-inflammatory cytokines and inflammatory mediators by regulating inflammation-related pathways, as shown in Table 5. The specific molecular pathways involved are depicted in Figure 3.

**TABLE 5:** The mechanisms involved in the anti-inflammation effect of HG and its derivatives.

Compound	Concentration	Model	Disease	Molecular mechanisms	References
HG	10, 30, 100 μM	LPS-induced RAW 264.7 cells	Not applicable	Inhibited the NF-κB signaling pathway	Lee et al. (2015)
HG	20 mg/kg	Rat model of atherosclerosis induced by high-fat diet and vitamin D_3_	Atherosclerosis	Suppressed the IKK/NF−κB signaling pathway	Lu et al. (2015)
Derivative 14	1, 3, 9 mg/kg	Mouse model of sepsis with acute liver injury induced by LPS	Acute liver injury in sepsis	Inhibited the STING/IRF3/NF-κB signaling	Yu et al. (2023)
Derivative 15	1.5, 2.5, 5 mg/kg	Mouse model of LPS-induced acute lung injury	Acute lung injury	Inhibited the STING/IRF3/NF-κB signaling	Li H. et al. (2024a)
HG	12.5, 25, 50 mg/kg	Rat model of sepsis-induced acute lung injury evoked by cecal ligation and puncture	Acute lung injury	Repressed NLRP3 inflammasome activation and M1 macrophage polarization and inhibited the NF-κB signaling pathway	Wang and Zhao (2022)
HG	50 mg/kg	Mouse model of type-2 diabetes	Type-2 diabetes	Suppressed the NF-κB and Smads signaling pathways	Li et al. (2019b)
HG	10 mg/kg	Rat model of neuropathic pain induced by a unilateral loose ligation of the sciatic nerve	Neuropathic pain	Decreased the phosphorylation of p38 MAPK	Zhang et al. (2020)
HG	50 mg/kg	Rat model of alcoholic liver disease	Alcoholic liver disease	Decreased p38 MAPK phosphorylation and increased phosphorylated AKT and ERK	Kim et al. (2017b)
HG	6.25, 12.5 μM	C28/I2 cells stimulated by IL-1β	Osteoarthritis	Suppressed the JAK2/STAT3 signaling pathway and MAPK signaling pathway and crosstalk between these two pathways, activated the Keap1-Nrf2/HO-1 signaling pathway	Shen et al. (2023)
HG	20, 40 mg/kg	Mouse model of acute kidney injury induced by cisplatin and LPS-induced renal tubular epithelial cells	Acute kidney injury	Inhibited the lncRNA A330074k22Rik/Axin2/β-catenin signaling pathway	Xie et al. (2022a)

The anti-inflammatory activity of a compound is directly related to its ability to inhibit the production of inflammatory mediators. Some studies have shown that HG can reduce the generation of inflammatory mediators, thus highlighting its anti-inflammatory effect. NO is an inflammatory mediator that is produced during the inflammatory process, and its excessive expression further exacerbates inflammatory injury (Wang Y. et al., 2019b). In experiments using RAW 264.7 macrophages, Zhang et al. discovered that HG reduced NO production (IC_50_ value = 22.26 μM) induced by lipopolysaccharide (LPS) (Zhang et al., 2012). In another study, HG exhibited anti-inflammatory effects by inhibiting NO and 15-lipoxygenase (15-LOX) generation, with IC_50_ values of 137.78 and 28.20 μM, respectively, in the same LPS-stimulated RAW 264.7 cells (Mfotie Njoya et al., 2023). The key enzyme 15-LOX participates in the biosynthesis of leukotrienes, which are essential mediators of the inflammatory response (Grosse et al., 2022).

#### 2.2.1 NF-κB

NF-κB, a heterodimer of p65 and p50, induces a cellular inflammatory response. In the resting state, NF-κB is sequestered in the cytoplasm by binding to its inhibitor, the inhibitor of kappa Bα (IκBα). Following stimulation, inhibitory-kappa B kinase (IKK) phosphorylates IκBα, resulting in IκBα degradation by proteasomes, the nuclear translocation of NF-κB, and the activation of inflammatory genes targeted by NF-κB (Liu and Su, 2023). Recent studies have shown that HG and its derivatives exert anti-inflammatory actions by blocking the NF-κB signaling pathway.

Lee et al. reported that HG suppressed the expression of inflammatory mediators and pro-inflammatory cytokines, including NO, prostaglandin E_2_ (PGE_2_), tumor necrosis factor-α (TNF-α), interleukin-1β (IL-1β), and IL-6, in LPS-induced RAW 264.7 cells. The underlying mechanism was related to inhibition of the NF-κB signaling pathway manifesting as the repression of IκBα phosphorylation and NF-κB nuclear translocation (Lee et al., 2015). In a rat model of atherosclerosis, HG exerted an anti-inflammatory effect by impeding the IKKβ/NF-κB signaling pathway and decreasing the levels of the pro-inflammatory cytokines IL-6, interferon-γ (IFN-γ), and TNF-α (Lu et al., 2015).

Upon detection of exogenous DNA within the cytoplasm, stimulator of interferon genes (STING) forms a complex with TANK-binding kinase 1 (TBK1), resulting in the activation of interferon regulatory factor 3 (IRF3) and NF-κB and an increase in the production of pro-inflammatory cytokines (Balka et al., 2020). In a mouse model of sepsis with acute liver injury induced by LPS, the new HG derivative **14** (Figure 1), with a pyrazine ring bonded to ring A, suppressed the levels of alanine aminotransferase (ALT), aspartate aminotransferase (AST), and alkaline phosphatase (ALP), attenuated the inflammatory response, and protected against liver injury. These effects were accomplished by inhibiting the level of STING, suppressing the phosphorylation of IRF3, TBK1, p65, and IκBα, and reducing the release of NO and pro-inflammatory cytokines including IL-6, TNF-α, and IFN-β. From the above results, it was suggested that the derivative inhibited inflammation by downregulating the STING/IRF3/NF-κB signaling (Yu et al., 2023). Recently, 33 hederagonic acid derivatives were designed and synthesized using HG as the fundamental building block. Among them, derivative **15** (Figure 1), carrying chained disulfide bonds at C-23, exhibited the greatest inhibition of the production of NO, thereby demonstrating potent anti-inflammatory activity. In a mouse model of LPS-induced acute lung injury (ALI), this derivative effectively prevented ALI by inhibiting the synthesis of pro-inflammatory cytokines, including TNF-α, IFN-β, and IL-6. The mechanism of action in part involved inhibition of the STING/IRF3/NF-κB signaling pathway (Li H. et al., 2024).

In a rat model of sepsis-induced ALI, which was established by cecal ligation and puncture, HG ameliorated septic lung injury, reduced the production of pro-inflammatory cytokines (TNF-α, IL-6, and monocyte chemoattractant protein-1), and decreased the levels of inducible nitric oxide synthase (iNOS) and cyclooxygenase-2 (COX-2). Additionally, HG significantly lowered NOD-like receptor pyrin domain-containing 3 (NLRP3), cleaved caspase-1, IL-1β, and IL-18. Interestingly, HG treatment accentuated the inhibitory effect of the NF-κB suppressor BAY11-7082 on NLRP3 inflammasome activation and M1 macrophage polarization *in vitro*. This study suggested that HG exerted an anti-inflammatory effect through inhibiting NLRP3 inflammasome activation and macrophage M1 polarization, which was partially linked to suppression of the NF-κB pathway (Wang and Zhao, 2022). In a type 2 diabetic mouse model, HG prominently inhibited myocardial hypertrophy and fibrosis and enhanced cardiac function in hearts of diabetic cardiomyopathy. The potential mechanisms involved were associated with inhibition of the NF-κB and Smads signaling pathways and a reduction in the levels of pro-inflammatory cytokines, including TNF-α, IL-1β, and IL-6 (Li Y. et al., 2019).

#### 2.2.2 MAPK

The mitogen-activated protein kinase (MAPK) pathway is related to induction of the inflammatory response and cell apoptosis. The MAPK family comprises three members: the c-Jun NH2-terminal kinase (JNK), the extracellular signal-regulated kinase (ERK), and p38 MAPK (Saleem, 2024). HG can effectively suppress activation of the MAPK signaling pathway, thus inhibiting inflammation and cell apoptosis, which renders it a promising therapeutic approach for various inflammatory diseases.

In a rat model of sciatic nerve injury caused by chronic constriction damage, HG alleviated sciatica by downregulating the expression of the pro-inflammatory cytokine TNF-α, attenuating the levels of transient receptor potential pathway proteins, and decreasing the phosphorylation of p38 MAPK (Zhang et al., 2020). In an alcoholic liver disease rat model, HG significantly protected against alcoholic liver damage, as demonstrated by the decreases in ALT and AST levels and the attenuation of liver fibrogenesis and inflammatory cell infiltration. In addition, HG exhibited not only an anti-inflammatory effect through inhibiting the production of pro-inflammatory cytokines TNF-α and IL-6, but also an anti-apoptotic effect by upregulating the expression of Bcl-2 and downregulating the expression of Bax and p53. The mechanisms involved included a decrease in p38 MAPK phosphorylation and an increase in the phosphorylation of AKT and ERK, which are two inhibitors of apoptosis (Kim G. J. et al., 2017).

The Janus kinase 2 (JAK2)/STAT3 signaling pathway plays a role in the initiation and progression of inflammatory responses, oxidative stress, and cell apoptosis, and its activation contributes to the erosion of cartilage (Chen et al., 2023). The Kelch-like ECH-associated protein 1 (Keap1)-Nrf2/heme oxygenase-1 (HO-1) pathway acts against oxidative stress and cell apoptosis, and can protect chondrocytes from oxidative and apoptotic responses (Pan et al., 2019). In a cell osteoarthritis model using IL-1β-stimulated C28/I2 cells, HG was reported to inhibit inflammation and protect cartilage by attenuating the increases in TNF-α, IL-6, iNOS, COX-2, NO, and PGE_2_, suppressing the JAK2/STAT3 and MAPK (JNK, ERK, p38) signaling pathways and crosstalk between these two pathways, and activating the Keap1-Nrf2/HO-1 signaling pathway. Notably, HG exerted an anti-apoptotic effect by suppressing ROS, downregulating Bax and cleaved caspase-3, and upregulating Bcl-2. The anti-inflammatory and chondroprotective effects of HG were also confirmed in an osteoarthritis rat model induced by monosodium iodoacetate (Shen et al., 2023).

#### 2.2.3 LncRNA A33/Axin2/β-catenin

The regulatory functions of lncRNAs in acute kidney injury (AKI) have been revealed in previous studies (Xie Z. et al., 2022b; Zhou et al., 2022). Axis inhibition protein 2 (Axin2)/β-catenin is recognized as an important contributor to the development and advancement of inflammation (Huang and Li, 2020). HG displayed a renoprotective effect in a cisplatin-induced mouse model of AKI by significantly inhibiting renal structural injury and decreasing serum creatinine and urea nitrogen levels. The underlying mechanism involved ameliorating the inflammatory responses via downregulation of the pro-inflammatory cytokines IL-6 and TNF-α and inhibition of the lncRNA A330074k22Rik (lncRNA A33)/Axin2/β-catenin signaling pathway *in vitro* and *in vivo* (Xie K. H. et al., 2022a). These findings indicate that HG can relieve AKI, which is a frequent side effect of the chemotherapeutic agent cisplatin.

#### 2.2.4 Immune regulation-related mechanisms

The complement system is a crucial part of the innate immune system, and excessive activation of this system underlies many inflammatory diseases (Guan et al., 2022). Therefore, suppressing complement activation can be of great use in the treatment of complement hyperactivation-associated inflammatory diseases. *In vitro*, HG exhibited anti-complementary activity by suppressing the classical complement activation pathways, with an IC_50_ value of 160 μM (Oh et al., 1999). In addition, a study confirmed that HG inhibited the generation of ROS (IC_50_ = 51.62 μM) from whole blood phagocytes activated by zymosan, thereby demonstrating its innate immune-suppressive effect, which contributes to the inhibition of detrimental immune reactions (Oladimeji et al., 2017). In atopic dermatitis models, HG-coated maghemite nanoparticles attenuated the expression of inflammation-related cytokines (TNF-α, IFN-γ, IL-4, IL-6, IL-17, and thymic stromal lymphopoietin) *in vitro* and *in vivo*. Moreover, HG decreased the severity of skin lesions, inhibited the infiltration of mast cells, and shrank enlarged lymph nodes by regulating the excessive immune response *in vivo*. These results clearly showed the immune-regulatory and anti-inflammatory effects of maghemite nanoparticles loaded with HG (Lee et al., 2022).

### 2.3 Infectious diseases

Infectious diseases include bacterial diseases, viral diseases, and parasitic diseases. Natural products are an essential source for the discovery of anti-pathogen medications. HG and its derivatives display broad-range anti-pathogen effects against bacteria, viruses, *Leishmania*, *Toxoplasma gondii* (*T. gondii*), and *Fasciola hepatica* (*F. hepatica*) (Table 6).

**TABLE 6:** The involved mechanisms in the anti-pathogen, anti-metabolic disorder, anti-fibrosis, neuroprotection, and anti-depression effects of HG and its derivatives.

Compound	Concentration	Model	Disease	Molecular mechanisms	References
Anti-pathogen					
HG	67.7 μM	*S. pneumoniae*	Pneumococcal diseases	Suppressed PLY oligomerization	Ding et al. (2022)
Derivatives 16, 17	1 μM	Hepatitis B virus	Hepatitis B	Inhibited the secretory expression of HBsAg	Hong et al. (2017)
Anti-metabolic disorder					
HG	9, 27, 81 mg/kg	Rat model of hyperlipidemia induced by a high-fat diet	Hyperlipidemia	Downregulated the p38 MAPK signaling pathway	Yang et al. (2022)
HG	0.1, 1, 10 μM	Mouse osteoblast MC3T3-E1 cells	Osteoporosis	Upregulated the expression of ALP, Runt-related transcription factor 2, and collagen Iα1	Huai et al. (2021)
HG	10 μM	Murine bone marrow macrophages	Osteoporosis	Constrained the formation of ROS and suppressed the MAPK signaling pathway	Tian et al. (2020)
HG	5 μM	T_84_ colonic epithelial cells, murine enteroid-derived monolayers	Intestinal and metabolic disorders	Upregulated the mRNA expression of FXR	Fallon et al. (2022)
Anti-fibrosis					
HG	20, 50 mg/kg	Rat model of pulmonary fibrosis induced by bleomycin	Pulmonary fibrosis	Downregulated the Ras/JNK/NFAT4 signaling pathway	Ma et al. (2020)
HG	Not mentioned	Hepatic stellate Lx2 cells and hepatic parenchymal L02 cells	Liver fibrosis	Increased PI3K expression	Wu R. et al. (2019a)
HG	5, 10, 15 μM	Fibroblasts NRK-49 F cells induced by TGF-β	Renal fibrosis	Suppressed the M3 muscarinic acetylcholine receptor	Yang and He (2022)
HG	25, 50 mg/kg	Mouse models of CKD induced by ischemia reperfusion injury and unilateral ureteral obstruction	Renal fibrosis	Inhibited ISG15 and restricted the JAK/STAT signaling pathway	Jia et al. (2023)
HG	2.5, 5, 10 μM	Human renal mesangial cells and human renal proximal tubular epithelial cells induced by high glucose	Renal fibrosis	Suppressed the activation of the NLRP3 inflammasome through decreasing cathepsin B expression	Yang et al. (2023)
HG	50 mg/kg	Mouse model of type-2 diabetes	Cardiac fibrosis	Suppressed the NF-κB and Smads signaling pathways	Li et al. (2019b)
Neuroprotection					
HG	12.5, 25, 50 mg/kg	Mouse model of middle cerebral artery occlusion	Cerebral ischemia/reperfusion injury	Decreased the activation of the MLK3 signaling pathway	Yu et al. (2020)
HG	25, 50 mg/kg	APP/PS1 transgenic mice	Alzheimer’s disease	Promoted PPARα/TFEB-dependent autophagy	Xie et al. (2023b)
HG	10 μM	Aβ-stimulated mouse hippocampal cell line HT22 cells and primary neuronal cells isolated from mouse hippocampus	Alzheimer’s disease	Promoted the activation of the PTPN1/AKT signaling	Li et al. (2023)
HG	60 μM	PC-12 cells	Parkinson’s disease and Huntington’s disease	Activated autophagy via the AMPK-mTOR signaling pathway	Wu et al. (2017)
Anti-depression					
HG	0.3 μM	PC12 cells injured by CORT	Depression	Increased MMP and reduced intracellular ROS accumulation via stimulation of the PI3K/AKT pathway	Lin et al. (2021)
HG	5 mg/kg	Rat model of unpredictable chronic mild stress-induced depression	Depression	Enhanced the mRNA expression of the 5-HT 1A receptor and reduced the mRNA expression of the 5-HT transporter	Liang et al. (2015)

#### 2.3.1 Bacterial diseases

Recent studies have highlighted the excellent anti-bacterial properties of HG *in vitro*. HG could moderately inhibit the growth of *Enterococcus faecalis* (*E. faecalis*) with a minimum inhibitory concentration (MIC) of 270.78 μM (Kayangar et al., 2019). *E. faecalis* is a refractory antibiotic-resistant bacterium that usually exists in biofilms (Sharma and Khan, 2018). Therefore, future research should focus on determining whether HG has anti-biofilm effects, and the specific molecular mechanisms underlying its anti-bacteria and possible anti-biofilm effects should be fully elucidated.

*Streptococcus pneumoniae* (*S*. *pneumoniae*) is an opportunistic bacterial pathogen that may lead to life-threatening diseases, such as pneumonia, meningitis, and sepsis, owing to its increased resistance to antibiotics (Fernandes et al., 2020). One of the virulence factors of this pathogen is pneumolysin (PLY), which induces host cell lysis following the formation of oligomeric proteins on the host cell membrane surface (Song et al., 2017). Ding and colleagues revealed that HG substantially inhibited the hemolytic activity and cellular injury induced by PLY *in vitro* through suppressing PLY oligomerization dose-dependently (Ding et al., 2022). Although HG did not significantly inhibit the growth of *S*. *pneumoniae*, it could prevent bacterial invasion and damage to the host via PLY inhibition. These observations illustrate that the natural product HG may amplify the effect of conventional antibiotic therapies in combating bacterial diseases, potentially helping to tackle the issue of antibiotic resistance.

#### 2.3.2 Viral diseases

Viral hepatitis is a profound global threat to human health. Some new HG derivatives have been confirmed to exhibit anti-viral hepatitis effects. Eight derivatives were synthesized by performing acetylation, etherification, benzoylation, and esterification reactions on the hydroxyl at C-3 and C-23, and the carboxyl at C-28 of HG. *In vitro*, seven out of the eight compounds significantly repressed the viability of Hep G2.2.15 cells in a dose-dependent fashion. Particularly, derivatives **16** and **17** (Figure 1) exhibited the most pronounced anti-hepatitis B virus effect, with an inhibition rate of 9.46% at a concentration of 1 μM. In addition, all of them could inhibit the secretory expression of HBsAg (Hong et al., 2017). Through a stepwise esterification reaction, 5 HG-hemiester-zidovudine derivatives were obtained, and derivative **18** (Figure 1) displayed the most potent inhibitory effect against the hepatitis C virus NS3/4A protease (IC_50_ = 5.8 μM) (Hao et al., 2021). These investigations verify that the natural compound HG is a promising source of virus inhibitors, and establish a foundation for subsequent detailed *in vivo* studies.

In a recent study targeting SARS-CoV-2 with *in silico* methods, HG presented an exceptionally high binding affinity with IL-6, a COVID-19-responsible receptor (Sai Ramesh et al., 2023). This interaction relationship suggests that HG may be an effective anti-SARS-CoV-2 agent. However, additional research and clinical trials are needed to validate this result.

#### 2.3.3 Parasitic diseases

##### 2.3.3.1 Leishmaniasis

Leishmaniasis, a series of sandfly-borne parasitic diseases, results from infection by *Leishmania* protozoan parasites (Cotton et al., 2020). HG and its derivatives have been shown to exhibit antileishmanial properties against many kinds of *Leishmania* species. The anti-leishmanial activity of HG was evaluated *in vitro* with promastigote and amastigote forms of *Leishmania infantum* (*L. infantum*) and *Leishmania tropica*. HG exhibited leishmanicidal activity against both the promastigote (MIC = 52.89 μM) and amastigote forms (Majester-Savornin et al., 1991). Among the 60 C-28-modified HG derivatives, 11 derivatives demonstrated apparent inhibition of the proliferation of *L. infantum* amastigotes. Derivatives **19–22** (Figure 1) were especially promising candidates for treating leishmaniasis due to their excellent leishmanicidal activity with IC_50_ values varying from 2 to 12 μM, high selectivity, and low toxicity (Rodríguez-Hernández et al., 2016a). Moreover, another study confirmed that structural transformation could increase the selectivity of the anti-leishmanial activity of the parent HG. In particular, derivative **23** (Figure 1), HG disuccinate, is a non-toxic anti-leishmanial derivative with high selectivity, and its activity warrants further investigation, because it is inactive against *Leishmania* amastigotes within the macrophage host (Anderson et al., 2020). The possible mechanisms underlying the antileishmanial activities of HG and its derivatives are summarized below.

###### 2.3.3.1.1 DPCK

Coenzyme A, an essential cofactor for numerous enzymes, plays a crucial role in various biochemical processes of *Leishmania*, and its synthesis requires dephospho-coenzyme A-kinase (DPCK) (Gudkova et al., 2012). The inhibition of DPCK is useful for treating leishmaniasis. A study revealed that HG interfered with the proliferation of *Leishmania donovani* promastigote cells *in vitro* (IC_50_ = 23.36 μM), potentially via DPCK suppression. Meanwhile, HG exhibited significant cytotoxicity against the J774A.1 murine macrophage line (Menpadi et al., 2023). The anti-leishmanial mechanism was validated only through *in silico* methods in the study, so further research is necessary.

###### 2.3.3.1.2 CYP51

Sterol 14a-demethylase (CYP51) is an enzyme essential for the viability and virulence of *Leishmania*, mainly participating in the biosynthesis of ergosterol. Interference with this biosynthetic process can disintegrate the cellular membrane and arrest the growth of *Leishmania* (McCall et al., 2015). By adding a triazolyl group at C-23 and C-28 of HG, 18 derivatives were designed and synthesized. Derivatives **24–26** (Figure 1) efficiently inhibited the multiplication of intracellular amastigote forms of *L. infantum* with IC_50_ values ranging from 5.6 to 25.9 μM, showing the highest potency and selectivity. The mechanisms of the leishmanicidal activity of derivatives 24–26 may include inhibition of the ergosterol biosynthesis pathway, as determined by molecular modeling. According to the results of molecular docking, there was strong binging affinity between the three bis-triazolyl derivatives and CYP51. This binding may lead to inhibition of the CYP51 enzyme by changing the structure of the active site (Rodríguez-Hernández et al., 2017).

##### 2.3.3.2 Toxoplasmosis

Toxoplasmosis, a zoonotic parasitic disease caused by the pathogen *T. gondii*, can damage the central nervous system and cause systemic disseminated infection in individuals with a compromised immune system (Hill et al., 2005). HG was found to exhibit robust anti-*T. gondii* activity (EC_50_ = 547.8 μM), high selectivity, and little cytotoxicity *in vitro*. In a mouse model of acute *T. gondii* infection, in addition to inhibiting *T. gondii* activity, HG also ameliorated *T. gondii*-induced liver injury, which was reflected by reducing the hepatotoxicity indicators (ALT and AST), lowering liver-damaging malondialdehyde (MDA), and increasing liver-protecting GSH (Zhang R. H. et al., 2021). However, the exact anti-toxoplasmic mechanism of HG remains obscure and needs to be uncovered. These findings imply that HG has the potential to be a part of the combination therapy regimen for toxoplasmosis, and the synergistic effect needs to be further validated.

##### 2.3.3.3 Fascioliasis

Infection with *F. hepatica* leads to fascioliasis, an important but neglected zoonotic disease. The appearance of drug-resistant *F. hepatica* poses a grave risk to animal and human health (Cwiklinski et al., 2016). A total of 36 compounds were synthesized through structural alterations of HG. Derivative **27** (Figure 1), which carries a morpholine ring on the ureate moiety, demonstrated noteworthy anthelmintic activity and selectivity against both immature and mature adult stage liver flukes (IC_50_ = 1.07 and 13.02 μM, respectively) (Chakroborty et al., 2023). Due to the notable anti-fluke activity exhibited by this derivative, its utilization could contribute to the mitigation of anthelmintic drug resistance.

### 2.4 Metabolic diseases

In recent years, there has been a significant global impact on human health due to the prevalence of metabolic diseases. Existing metabolic regulators have corresponding adverse effects, such as damage to liver and kidney function. Natural products, which have less harmful side effects than synthetic drugs, are gradually becoming a significant focus of research on the treatment of metabolic disturbances. Fortunately, HG has been shown to regulate abnormal substance metabolism (Table 6).

#### 2.4.1 Hyperglycemia

As a complex metabolic disease, diabetes mellitus (DM) is featured by chronic hyperglycemia and multisystemic involvement. Due to the protracted course of the disease and numerous complications, DM can cause severe damage to human health, and subsequently brings heavy societal economic burdens (Ren et al., 2022). In a classical rat model of diabetes induced by streptozotocin, HG was preliminarily observed to reverse the elevation in the serum glucose level, which was reduced by roughly one-third, conferring a modest hypoglycemic effect (Park et al., 1998).

##### 2.4.1.1 Glycogen phosphorylase

During glucose metabolism, glycogen phosphorylase (GP) is essential for the breakdown of glycogen into glucose for energy production. This implies that the inhibition of GPs can facilitate glycemic control (Yan et al., 2022). HG at a concentration of 10 μM displayed significant GP inhibitory activity with a 73.07% suppression ratio (Luo et al., 2008).

##### 2.4.1.2 Starch digestion enzymes

The α-amylase and α-glucosidase enzymes are crucial for breaking down starch into glucose, leading to an elevation in blood sugar levels. HG demonstrated excellent inhibitory activity against both α-amylase and α-glucosidase, with an IC_50_ value of 162.89 μM *in vitro*, and alleviated postprandial hyperglycemia *in vivo* (Han et al., 2024). However, further in-depth investigations are required to determine the precise anti-hyperglycemic effect and mechanism of HG due to the limited number of studies addressing this issue.

#### 2.4.2 Hyperlipidemia

Hyperlipidemia is a major contributor to fatty liver disease, atherosclerosis, and cardio-cerebrovascular diseases (Ross, 1993). Coincidentally, the administration of HG is beneficial for improving the blood lipid profile. HG could effectively regulate the aberrant outlines of lipid metabolism elicited by DM in a rat model, as evidenced by reducing the levels of total cholesterol (TC), low-density lipoprotein (LDL)-cholesterol, and triglyceride (TG) and increasing the level of high-density lipoprotein (HDL)-cholesterol (Park et al., 1998). Consistently, a similar modulation of lipid levels was observed in a rat model of atherosclerosis. In this work, HG was also proven to have an anti-atherosclerosis effect by alleviating the disturbance in lipid metabolism and decreasing the deposition of lipids on vascular walls (Lu et al., 2015).

Moreover, an investigation focusing on the lipid-regulating mechanism of HG was recently published. Oxidative stress triggers hyperlipidemia (Masenga et al., 2023), and its inhibition can reverse the deranged lipid profile. MAPK is related to oxidative stress, and the overactivation of the MAPK pathway result in cellular oxidative damage (Rezatabar et al., 2019). In hyperlipidemic model rats treated with a high-fat diet, HG dramatically restored the levels of TC, TG, LDL-cholesterol, and HDL-cholesterol, reduced MDA, increased total superoxide dismutase (SOD) and GSH peroxidase, and inhibited p38 MAPK phosphorylation. Mechanistically, HG exerted a hypolipidemic effect by suppressing the absorption and oxidation of lipids and downregulating the p38 MAPK pathway (Yang et al., 2022).

#### 2.4.3 Osteoporosis

Osteoporosis is a bone metabolic derangement distinguished by bone tissue deterioration and bone mass reduction due to excessive bone resorption and inadequate bone formation (Eastell et al., 2016). Relevant studies have shown that HG can function as both an activator of bone formation and a suppressor of bone resorption, thus contributing to the maintenance of bone metabolic homeostasis.

##### 2.4.3.1 Facilitation of bone formation

ALP, Runt-related transcription factor 2 (RUNX2), and collagen type I α 1 chain (COL Iα1) are hallmark genes of osteogenic differentiation (Komori, 2010; Mostofi et al., 2022). With the MC3T3-E1 mouse osteoblast cell line, Huai and coworkers revealed that HG displayed an anti-osteoporosis effect based on facilitating the differentiation and proliferation of osteoblasts via upregulating the expression of the above osteoblast differentiation-related characteristic genes (Huai et al., 2021).

##### 2.4.3.2 Attenuation of bone resorption

ROS participate in the processes of osteoclastogenesis and bone resorption (Callaway and Jiang, 2015). The MAPK signaling pathway is additionally implicated in the formation of osteoclasts, whose inhibition suppresses osteoclastogenesis (Li et al., 2021). A study employing murine bone marrow macrophages demonstrated that HG inhibited osteoclastogenesis and bone resorption induced by receptor activator of NF-κB ligand through constraining the formation of ROS and suppressing the MAPK signaling pathway (p38 and ERK). *In vivo*, HG also hindered the formation and function of osteoclasts in a mouse model of osteoporosis induced by ovariectomy (Tian et al., 2020).

#### 2.4.4 Other associated mechanisms

##### 2.4.4.1 FXR

The farnesoid X receptor (FXR) is pivotal for the modulation of intestinal function and glucose and lipid metabolism processes. The effects of FXR activation and overexpression in metabolic diseases have been conclusively confirmed (Sonne, 2021). HG upregulated FXR mRNA expression, enhanced FXR agonist-induced FXR activation, and improved the anti-secretory function of FXR in colonic epithelial cells. These results indicated that HG might be effective in treating diseases associated with intestinal and metabolic disorders via targeting the FXR signaling pathway (Fallon et al., 2022).

##### 2.4.4.2 11β-HSD1

Excessive levels of glucocorticoids can lead to metabolic diseases such as type-2 diabetes, hypertension, dyslipidemia, and visceral obesity. 11β-Hydroxysteroid dehydrogenase type 1 (11β-HSD1) is an intracellular regulatory enzyme that regenerates active glucocorticoids from inactive inert 11-keto forms. Inhibition of 11β-HSD1 can ameliorate metabolic disturbances (Wamil and Seckl, 2007). Yan et al. determined that HG moderately inhibited mouse 11β-HSD1 *in vitro* (IC_50_ = 0.16 μM), thus preventing the over production of glucocorticoids (Yan et al., 2021).

##### 2.4.4.3 FAS

Fatty acid synthase (FAS) is a key enzyme that catalyzes the synthesis of fatty acids, which can be transported into adipocytes, ultimately inducing adiposity. FAS inhibition leads to repression of food intake and a decrease in body weight (Loftus et al., 2000). HG exhibited specific inhibitory activity against FAS (IC_50_ = 128.51 μM), indicating its potential application as a promising anti-obesity agent (Zhao et al., 2011).

The pathogenesis of metabolic diseases is intricate, and involves multiple targets, pathways, and systems. As outlined previously, HG can modulate the metabolic process of several substances through selectively targeting metabolism-related enzymes and genes. As an effective active component for modulating metabolic disorders, HG has the potential to be an ideal candidate for the treatment of metabolic disease. However, further clinical research is still needed due to the absence of data from humans.

### 2.5 Fibrotic diseases

Fibrotic disease is a category of progressive diseases hallmarked by increased fibrous connective tissue and aberrant deposition of the extracellular matrix, particularly collagens, eventually culminating in a decrease in parenchyma cells and organ function decline (Xing et al., 2021). However, effective therapies for patients with fibrosis are limited, and the search for potent therapeutic methods is sorely required. Surprisingly, HG has exhibited broad clinical application prospects for the treatment of fibrotic disorders (Table 6).

#### 2.5.1 Ras/JNK/NFAT4

Ras proteins are small GTPases, participating in the pathogenesis of pulmonary fibrosis (Liu et al., 2018). JNK contributes to fibrosis by facilitating the epithelial-mesenchymal transition (EMT) (Alcorn et al., 2009), and it can stimulate nuclear factor of activated T cells 4 (NFAT4) to regulate the expression of related genes (Chow et al., 1997). In a bleomycin-induced pulmonary fibrosis rat model, HG relieved pulmonary function impairment and pathological lesions in a dose-dependent manner. In addition, HG alleviated the deposition of collagen marked by a reduction in the levels of hydroxyproline, α-smooth muscle actin (α-SMA), and collagen I, inhibited EMT manifested by a decrease in transforming growth factor-β1 (TGF-β1), and suppressed the levels of inflammatory cytokines TNF-α and IL-6. The anti-pulmonary fibrosis effect of HG was associated with inhibition of the Ras/JNK/NFAT4 signaling (Ma et al., 2020).

#### 2.5.2 PI3K

The PI3K-AKT pathway hampers apoptosis and provokes proliferation (Xue et al., 2020), and its low expression can result in the apoptosis of hepatic parenchymal cells (HPCs) in liver fibrosis. The balance between apoptosis and proliferation in HPC may be restored by increasing the expression of PI3K. With the hepatic stellate cell (HSC) line Lx2 and the HPC line L02, HG exerted an anti-liver fibrosis effect by inhibiting the activation of HSCs and reducing the levels of α-SMA and collagen I, as well as protecting HPCs from apoptosis (reflected by the decline in the ratio of Bax/Bcl-w and the expression of cleaved caspase-3). The apoptosis-suppressing mechanism of HG in HPCs was mediated by an increase in PI3K expression (Wu et al., 2019a).

#### 2.5.3 Muscarinic acetylcholine receptor

The participation of the M3 muscarinic acetylcholine receptor (M3 receptor) in the production of collagen and fibroblast proliferation renders it a potential drug target for fibrotic diseases (Zhao et al., 2019). By exploiting TGF-β-stimulated fibroblast NRK-49 F cells to simulate renal fibrosis, Yang et al. reported that HG was able to inhibit the proliferation and fibrosis of NRK-49 F cells, decrease the expression of α-SMA, collagen I, and collagen III, and downregulate the level of phosphorylated Smad2/3 through suppressing the activity of the M3 receptor (Yang and He, 2022).

#### 2.5.4 ISG15

Interferon-stimulated gene 15 (ISG15), a ubiquitin-like protein, is implicated in various intracellular processes, such as apoptosis, autophagy, and signal transduction (Zhao et al., 2005). ISG15 can trigger the JAK/STAT pathway, and activation of this pathway has the potential to worsen renal fibrosis (Liu Y. et al., 2020; Nakka and Mohammed, 2020). HG was shown to exert significant anti-renal fibrosis and nephroprotective effects by ameliorating structural damage in the kidney and decreasing the fibrosis-related proteins fibronectin and α-SMA in two mouse models of chronic kidney disease (CKD). Mechanistically, HG administration promoted the regression of fibrosis in CKD by restricting ISG15 activity and its downstream JAK/STAT signaling pathway (Jia et al., 2023).

#### 2.5.5 Cathepsin B

Cathepsin B, a lysosomal protease, is crucial for initiating NLRP3 inflammasome activation and fibrosis under specific circumstances (Bai et al., 2018; Sung et al., 2019). Using a cell model of diabetic nephropathy induced by high glucose, Yang et al. discovered that HG prevented renal cell fibrosis by reducing the elevated levels of fibronectin, collagen IV, plasminogen activator inhibitor 1, and TGF-β1. The anti-renal fibrosis effect of HG was accomplished by suppressing activation of the NLRP3 inflammasome through decreasing cathepsin B expression (Yang et al., 2023).

#### 2.5.6 NF-κB and Smads

As a pivotal component of the inflammatory signaling pathway, NF-κB is capable of facilitating inflammatory fibrosis (Zhang Y. et al., 2022b). The Smad signaling pathway induced by TGF-β is crucial for the formation and progression of fibrosis (Xu et al., 2017). In a mouse model of type-2 diabetes, HG significantly reduced myocardial fibrosis caused by diabetes and inhibited collagen I and TGF-β1 synthesis in cardiomyocytes, thereby exhibiting anti-cardiac fibrosis properties via inhibiting the NF-κB and Smads signaling pathways (Li Y. et al., 2019b).

These studies showed that HG can mitigate various fibrotic conditions, including pulmonary fibrosis, liver fibrosis, renal fibrosis, and cardiac fibrosis. Therefore, HG demonstrates promise as a feasible strategy for the development of novel pharmaceutical interventions aimed at preventing fibrosis. Further investigation is necessary to substantiate the clinical efficacy and safety of HG in humans prior to its implementation in clinical practice.

### 2.6 Cerebrovascular and neurodegenerative diseases

The application of neuroprotective agents is imperative to effectively safeguard neuronal function during the treatment of cerebrovascular and neurodegeneration-related disorders. HG exhibits promising potential as a natural candidate for neuroprotection (Table 6).

#### 2.6.1 Ischemic stroke

Ischemic stroke is a cerebrovascular disease characterized by the occlusion of cerebral blood vessels within specific brain regions (Haupt et al., 2023). The shortage of efficacious neurologically protective drugs is a significant challenge for dealing with ischemic stroke. However, administration of HG may yield the desired outcomes.

Following cerebral ischemia, *S*-nitrosylation of mixed lineage kinase 3 (MLK3) promotes its activation, which influences the phosphorylation and activation of its downstream proteins (Hu et al., 2012). Yu et al. investigated the neuroprotective effect of HG via a mouse model of middle cerebral artery occlusion. HG decreased cerebral infarction volumes and alleviated neurological impairments by inhibiting neuronal apoptosis and the inflammatory response. The potential mechanism might involve repression of the MLK3 signaling pathway, which results in the downregulation of its downstream signaling pathways, namely, MAPK and NF-κB pathways (Yu et al., 2020).

#### 2.6.2 Alzheimer’s disease

Alzheimer’s disease (AD) is characterized by a gradual decline in cognitive function, and currently, the drugs available for treatment are limited to providing symptomatic relief. HG demonstrates potential for the treatment of AD by targeting amyloid β (Aβ) and acetylcholinesterase (AChE).

##### 2.6.2.1 Aβ

The primary feature of AD is the presence of extracellular senile plaques that are composed of Aβ peptides. Among these peptides, Aβ42 is particularly fibrillogenic and has a greater propensity to induce the formation of protofibrils and fibrils, ultimately leading to the development of senile plaques (Selkoe, 2001). At a concentration of 40 μM, HG was discovered to have a significant inhibitory effect on Aβ42-induced fibrillogenesis (Chowdhury et al., 2017). The exacerbation of Aβ aggregation in AD pathogenesis is observed when there is a disruption in autophagic flux (Zhang W. et al., 2022a). As a member of the ligand-activated nuclear receptor superfamily, peroxisome proliferator-activated receptor α (PPARα) can induce the upregulation of transcription factor EB (TFEB) expression, which promotes autophagy through positively modulating the expression of genes associated with lysosomes and autophagy (Luo R. et al., 2020b). HG alleviated the deposition of Aβ and improved cognitive impairment by enhancing autophagy via activating the PPARα/TFEB pathway (Xie et al., 2023b).

Protein tyrosine phosphatase nonreceptor type 1 (PTPN1) can mitigate memory deficits and Aβ formation in models of AD (Wang et al., 2018b). AKT, which is related to AD repression, lies downstream of PTPN1 (Liang et al., 2023). By utilizing the Aβ-stimulated mouse hippocampal cell line HT22 cells and primary neuronal cells isolated from the mouse hippocampus to establish a cellular model of AD, HG alleviated the Aβ-induced increase in ROS accumulation and decline in SOD activity, and reduced the Aβ-induced elevation in the apoptotic rate and caspase-3 activity. The anti-oxidative stress and anti-apoptotic activities of HG were accomplished via promoting the activation of the PTPN1/AKT signaling (Li et al., 2023).

##### 2.6.2.2 AChE

AChE breaks down the neurotransmitter acetylcholine, which plays a crucial role in regulating the plasticity of synapses, the transmission of synaptic information, and the excitability of neuronal cells (Picciotto et al., 2012). Cognitive decline in AD is believed to be linked to cholinergic function deficiency (Haam and Yakel, 2017). *In vitro*, HG was proven to exhibit a general suppressive capacity (IC_50_ = 278.80 μM) against AChE to potentiate cholinergic function (Zhang W. et al., 2021).

#### 2.6.3 Parkinson’s disease and Huntington’s disease

In addition to AD, Parkinson’s disease and Huntington’s disease are two other neurodegenerative disorders that are characterized by the formation and aggregation of neurotoxic mutated proteins (Rubinsztein, 2006). The induction of autophagic degradation is crucial for the elimination of abnormally accumulated mutant proteins. The adenosine-monophosphate-activated protein kinase (AMPK)-mammalian target of rapamycin (mTOR) signaling pathway is a key signaling involved in autophagy. The activation of autophagy-promoting AMPK encourages the restriction of autophagy-suppressing mTOR, resulting in enhanced autophagy (Li Y. Y. et al., 2024). HG could facilitate the degradation of mutant huntingtin with 74 CAG repeats and A53T α-synuclein and suppress huntingtin inclusion formation and the oligomerization of α-synuclein through activating the autophagy process via the AMPK-mTOR signaling pathway (Wu et al., 2017).

Based on the aforementioned analysis, the neuroprotective activity of HG primarily stems from its pro-autophagy, anti-apoptosis, anti-inflammatory, and anti-oxidative stress effects. However, it is still necessary to conduct meticulously designed clinical trials that can provide additional meaningful insights about HG for clinical applications and scientific investigations.

### 2.7 Depression

Depression is a widespread and serious psychological illness with the potential to be life-threatening. Nevertheless, existing antidepressants frequently come with significant adverse effects. Therefore, development of safe and effective medications for the treatment of depression is urgently needed. Several studies have focused on the potential anti-depression effect of HG (Table 6).

#### 2.7.1 HPA axis

Depression exhibits a strong association with the hyperactivity of the hypothalamic-pituitary-adrenal (HPA) axis, which results in the elevation of corticosterone (CORT) serum levels (Troubat et al., 2021). *Fructus Akebiae* extracts (FAE) containing approximately 70% HG improved motivational behavioral impairments, corroborating its anti-depression activity, the mechanism of which was through decreasing the excessive activity of the HPA axis, featured by reduced plasma adrenocorticotrophic hormone and serum CORT levels (Zhou et al., 2010). An additional study concluded that HG exerted an anti-depression role by protecting nerve cells against damage and apoptosis caused by elevated CORT level. In CORT-injured PC12 cells, HG ameliorated neuronal injury and apoptosis induced by CORT through blocking the decline in the MMP and decreasing the generation of intracellular ROS via stimulation of the PI3K/AKT pathway, which is essential for the survival and proliferation of neuronal cells (Lin et al., 2021).

#### 2.7.2 Monoamine neurotransmitters

The monoamine hypothesis posits that depression primarily arises from a deficiency in monoamine neurotransmitters, and enhancing monoamine neurotransmitter levels is a critical therapeutic approach for depression (Jiang et al., 2022). FAE with about 90% enrichment of HG could suppress the reuptake of central monoamine neurotransmitters, including serotonin (5-HT), norepinephrine (NE), and dopamine (DA) by inhibiting the 5-HT transporter, the NE transporter, and the DA transporter dose- and time-dependently (Jin et al., 2012). HG exhibited a more potent anti-depressive effect than FAE by increasing the levels of NE and 5-HT, enhancing the mRNA expression of the 5-HT 1A receptor, and reducing the mRNA expression of the 5-HT transporter (Liang et al., 2015).

Two of the hypotheses regarding the pathogenesis of depression are hyperactivation of the HPA axis and a deficiency of monoamine neurotransmitters. Correspondingly, the above results from studies of depression revealed that HG can hinder HPA axis activity and enhance signaling in the central monoaminergic system to alleviate depression. As a potentially effective therapeutic intervention for depression, further experimental and properly conducted clinical research is needed to elucidate the mechanism of the antidepressant action of HG.

### 2.8 Other effects and mechanisms of HG

#### 2.8.1 Anti-platelet aggregation

A preliminary investigation demonstrated the anti-platelet aggregating property of HG. HG exhibited an inhibitory effect on the platelet aggregation induced by epinephrine (IC_50_ = 32.8 μM), which was comparable to that of acetylsalicylic acid (IC_50_ = 57.0 μM) (Jin et al., 2004). Thus, further comprehensive animal testing is required to elaborate on the efficacy and mechanism of HG as a novel inhibitor of platelet aggregation, although it is highly probable that HG can effectively inhibit platelet aggregation to hinder the formation of thrombi.

#### 2.8.2 Inhibition of ferroptosis

When myocardial ischemia-reperfusion occurs, iron is deposited in cardiomyocytes, which damages lipid membranes by encouraging the synthesis of lipid peroxides, ultimately resulting in cardiomyocyte ferroptosis (Ding et al., 2023). In a study of myocardial ischemia-reperfusion injury, HG prevented ferroptosis in the myocardium and cardiomyocytes through decreasing lipid peroxidation and iron levels. The anti-ferroptosis effect of HG partially relied on the inhibition of 5-LOX, which is a key enzyme involved in the initiation of ferroptosis (Zhao et al., 2023).

## 3 Pharmacokinetics of HG and its derivatives

### 3.1 Pharmacokinetics of HG

Examining the pharmacokinetic profile of HG facilitates our understanding of the dynamic changes HG undergoes in the body, which is crucial for developing drugs and optimizing dosing methods. The oral absorption properties of HG were assessed using the ultra fast liquid chromatography-tandem mass spectrometry method. The study showed that HG was detectable in rat plasma within 5 min following a single administration of 400 mg/kg FAE (about 280 mg/kg HG), reaching a peak concentration (Cmax) of 47.73 ± 1.39 ng/mL approximately 20 min after oral administration. The elimination of HG occurred rapidly, with an elimination half-life (t1/2) of 44.06 ± 2.98 min (Yang X. et al., 2011). In addition, Zhang et al. evaluated the pharmacokinetics of HG in the plasma of rats subsequent to the oral administration of *Rhizoma Clematidis* extract. The pharmacokinetic parameters after the administration of 35 g/kg *Rhizoma Clematidis* extract (equivalent to 232 mg/kg HG) were as follows: Cmax = 30.68 ± 4.32 ng/mL, time to reach the maximum concentration (Tmax) = 21.67 ± 7.53 min, and t1/2 = 50.87 ± 21.26 min (Zhang et al., 2017), which showed noticeable consistency with the former research. As shown by the two experiments, HG demonstrates rapid absorption and quick elimination after oral administration.

HG is a metabolite of asperosaponin VI. After oral administration of 90 mg/kg asperosaponin VI, HG was initially detected in rat plasma after 3 h. HG reached a Cmax of 25.5 ± 11.8 ng/mL at a Tmax of 13.0 ± 3.6 h, and the t1/2 was 5.6 ± 3.4 h (Zhu et al., 2011). Liu and colleagues achieved comparable outcomes through the oral administration of asperosaponin VI to rats at a dosage of 100 mg/kg. The Cmax for HG was 53.18 ± 23.27 ng/mL, and this concentration was reached at 12.33 ± 2.36 h (Tmax) (Liu et al., 2014b). Both studies indicate that the conversion of HG from asperosaponin VI may result in a comparative increase in bioavailability due to slow absorption and prolonged elimination time.

The tissue distribution of HG is restricted. An investigation of tissue distribution was conducted to measure the concentrations of asperosaponin VI and HG in 13 rat tissue samples following the intragastric administration of asperosaponin VI at a dosage of 270 mg/kg. The tissues examined included the kidney, brain, ovary, testis, liver, heart, spleen, lung, fat, muscle, stomach, large intestine, and small intestine. HG underwent predominant intestinal flora metabolism prior to plasma absorption, as it was not detectable in the majority of tissues with the exception of the gastrointestinal tract (Zhang et al., 2014). Significantly, after a single administration of 400 mg/kg FAE (about 280 mg/kg HG), HG was identified at a concentration of 6.17 ± 0.22 ng/mL in the cerebrospinal fluid of rats after 20 min. These findings indicate that HG can penetrate the blood-brain barrier and quickly spread into the cerebrospinal fluid, providing a material basis for the neuroprotective and antidepressant effects of HG (Yang et al., 2011a).

Research on drug metabolism plays a crucial role in discovering and developing drugs. Cytochrome P450 (CYP) enzymes are responsible for the metabolism of most drugs (Reed et al., 2007). Therefore, it is vital to explore the effects of candidate drugs on the activities of CYP enzymes. *In vitro*, HG exhibited a particular inhibitory effect on CYP2C9 and CYP2C19, with IC_50_ values of 4.94 and 18 μM, respectively, in human liver microsomes (Huang et al., 2022a). In subsequent research on HG preparations, it should be noted that HG may competitively inhibit other drugs that interact with CYP2C9 and CYP2C19.

The preceding pharmacokinetic studies were conducted on HG-comprising plants or other compounds that can be converted into HG. This implies that the interaction between HG and other constituents may influence the pharmacokinetic features of HG. Further pharmacokinetic studies should be performed on HG as a single component.

### 3.2 Pharmacokinetics of HG derivatives

Improvements in the pharmacokinetic patterns of HG derivatives would be more meaningful for the research and development of drugs taking HG as the fundamental structure. In a study conducted by Ma et al., three groups of beagle dogs were administered orally with the same dose (30 mg/kg) of HG, asperosaponin VI, or HG sodium succinate. The results revealed that the oral bioavailability of HG sodium succinate was significantly superior to that of HG and asperosaponin VI. HG sodium succinate reached a Cmax of 4047.35 ± 943.96 ng/mL with a corresponding Tmax of 0.88 ± 0.35 h, and its t1/2 was 1.38 ± 0.53 h. HG was detected in a small part of samples and its concentrations were around the lower limit of quantification (5 ng/mL). The Cmax, Tmax, and t1/2 for asperosaponin VI were 196.81 ± 83.96 ng/mL, 1.54 ± 0.46 h, and 2.68 ± 1.08 h, respectively (Ma et al., 2021). However, pharmacokinetic research of HG derivatives is rare and should be further strengthened.

## 4 Safety, targeting, and bioavailability of HG and its derivatives

With the increasing recognition of the therapeutic benefits of HG and its derivatives, there has been growing concern regarding their safety, targeting, and bioavailability. Fortunately, several efforts have been made to resolve the concerned issues of these compounds.

### 4.1 Safety

HG was discovered to have side effects *in vitro* and *in vivo*. HG exhibited a moderate level of intrinsic hemolytic activity *in vitro*, which can be attributed to the polar carboxyl group at C-28. The association between structure and hemolytic activity above has yielded valuable insights for the advancement and manufacture of HG derivatives free from toxicity resulting from red blood cell hemolysis (Vo et al., 2017).

In breast cancer-bearing mice, administration of HG via tail vein injection resulted in no side effects, except for myelosuppression, which was characterized by a decrease in the levels of red blood cells and white blood cells. Remarkably, a novel nano-delivery system, a nanoplatform of platelet membrane-coated black phosphorus quantum dots loaded with HG, not only reduced the adverse effect of bone marrow inhibition caused by free HG but also potentiated the anti-cancer efficacy of HG through the facilitation of cell apoptosis and autophagy (Shang et al., 2019). In addition, the safety of HG was evaluated in a mouse model with SKOV3 ovarian tumor xenografts. HG caused no significant damage to vital organs covering the heart, liver, spleen, lung, and kidney (Su et al., 2024). The side effects and safety profiles of HG and its derivatives have yet to be completely explored. Therefore, further research is required.

### 4.2 Targeting

The poor targeting of HG limits its therapeutic efficacy. However, nanomedicine technology has been applied to improve its targeting ability. Aforementioned nanoplatform of black phosphorus quantum dots camouflaged with a platelet membrane exhibited superior drug-loading efficiency and compatibility. HG could be targeted for delivery to tumor tissue through this platform (Shang et al., 2019). Another secured drug vehicle, maghemite nanoparticles, which are non-toxic, stable, and biocompatible, could deliver HG precisely to the sites of inflammation, thereby improving the effectiveness and targeting of HG (Lee et al., 2022).

### 4.3 Bioavailability

The poor water solubility of HG and some of its derivatives results in restricted bioavailability. H6, which has poor solubility, is a synthetic derivative of HG. However, introducing nitrogen-containing groups at C-28 (Wang et al., 2019a) or PEG molecules at C-23 or C-28 (Wang et al., 2021) can efficiently increase the solubility and bioavailability of H6. According to pharmacokinetic analysis, the low bioavailability of HG may also be attributed to its rapid systemic elimination. In subsequent studies, more attention should be paid to alter the absorption, distribution, metabolism, and excretion characteristics of HG and its derivatives.

## 5 Conclusion and future perspectives

**FIGURE 4:**
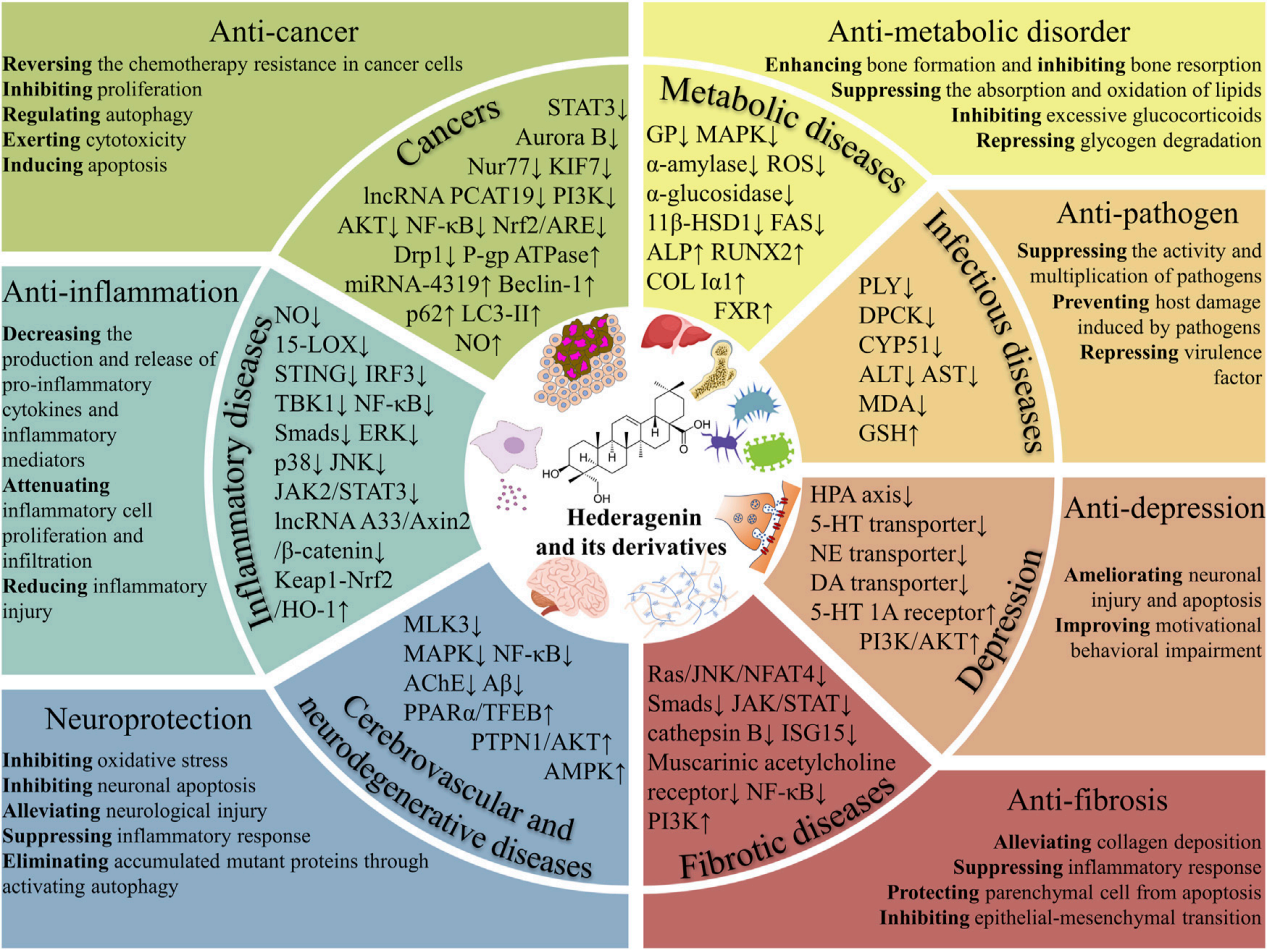
Extensive pharmacological effects and related action targets of HG and its derivatives.

This review focused on the action targets of HG and its derivatives that have been discovered in recent years. Figure 4 shows the action targets of HG and its derivatives to exhibit pharmacological activities against cancers, inflammatory diseases, infectious diseases, metabolic diseases, fibrotic diseases, cerebrovascular and neurodegenerative diseases, and depression.

The anti-cancer effects of HG and its derivatives have been studied in carcinomas of diverse systems, comprising lung cancer, liver cancer, oral cancer, gastric cancer, breast cancer, cervical cancer, ovarian cancer, thyroid cancer, colon cancer, HNC, and glioma. HG and its derivatives demonstrate anti-tumor effects mainly through cytotoxic activity against tumor cells, proliferation inhibition, apoptosis induction, autophagy regulation, and amelioration of chemotherapy resistance in cancer cells. HG suppresses the proliferation of tumor cells by inhibiting the STAT3 pathway, the Aurora B pathway, and the PI3K/AKT pathway, downregulating lncRNA PCAT19 and KIF7 expression, and upregulating miRNA-4319 expression. In addition, cancer cell apoptosis induced by HG and its derivatives is closely correlated with mitochondria-dependent intrinsic apoptotic pathways, which are activated by the release of ROS and cyt c from impaired mitochondria. The NF-κB pathway, the Nrf2/ARE pathway, and Drp1 are involved in the inducing effect of HG and its derivatives on cell apoptosis. The inhibition of these targets and pathways leads to upregulation of the apoptosis-promoting proteins Bad and Bax, downregulation of the apoptosis-suppressing proteins Bcl-2, Bcl-xL, and Survivin, an increase in caspase-9, caspase-3, and caspase-7 activity, and promotion of cleaved PARP, ultimately resulting in cell apoptosis in various cancers. Moreover, HG can not only activate tumor-suppressing autophagy by upregulating Beclin-1 expression to exert an anti-cancer effect but also hinder cell death preventative autophagy by inhibiting lysosomal acidification to potentiate the cytotoxicity of chemotherapeutic drugs. Furthermore, HG and its derivatives can effectively improve the chemotherapy resistance of tumor cells to strengthen the efficacy of chemotherapeutics through suppressing the drug efflux pump function of P-gp, boosting the level of NO, and blocking the PI3K/AKT pathway. Collectively, HG and its derivatives demonstrate anti-cancer effects by exerting cytotoxicity, restricting proliferation, stimulating apoptosis, modulating autophagy, and reversing chemotherapy resistance via complicated molecular mechanisms.

Additionally, HG and its derivatives have shown anti-inflammatory effects in a variety of diseases through reducing the generation and release of pro-inflammatory cytokines and inflammatory mediators, by inhibiting the NF-κB pathway, the MAPK pathway, the JAK2/STAT3 signaling pathway, and the lncRNA A33/Axin2/β-catenin pathway, and activating the Keap1-Nrf2/HO-1 signaling pathway. Moreover, HG and its derivatives exhibit anti-infective activities against a wide spectrum of pathogens, including bacteria, viruses, *Leishmania*, *T. gondii*, and *F. hepatica*. As a metabolism-regulating agent, HG can affect the metabolic processes of glucose, lipids, and bone. For instance, HG recovers abnormal bone metabolism by modulating the expression of osteoblast differentiation-related genes (ALP, RUNX2, and COL Iα1), inhibiting ROS formation, and suppressing the MAPK pathway. Impressively, HG has been found to possess anti-fibrotic effects through modulation of the Ras/JNK/NFAT4 pathway, the muscarinic acetylcholine receptor, the ISG15-modulated JAK/STAT signaling pathway, the expression of cathepsin B, the NF-κB pathway, and the Smads pathway. Moreover, HG can protect neuronal function to treat neurodegenerative and cerebrovascular diseases, improve depression, suppress aggregation of platelets, and prevent ferroptosis.

This review combed relevant research on the pharmacological effects and underlying mechanisms of HG and its derivatives, with disease targets as the main focus. Additionally, their drawbacks with regards to drug safety, targeting, and bioavailability were also discussed. Although structural modifications and nanomedicine technologies have been applied productively to ameliorate deficiencies and increase the therapeutic efficacy of HG and its derivatives, much work remains. In particular, available data regarding their side effects are extremely limited. Moreover, all existing studies on their pharmacological effects are limited to animal and cell experiments. Therefore, more experimental and clinical investigations into their medicinal safety and toxicity to determine precise dose-effect-toxicity relationships are highly warranted for developing HG and its derivatives as novel pharmaceuticals. In addition, introducing new preparation technologies and drug delivery systems is necessary to improve their safety, targeting, water solubility, and bioavailability. Another concern is that the mechanisms of some actions of HG and its derivatives remain opaque. More cellular and animal studies are required to reveal the relevant mechanisms involved in various diseases holistically. In conclusion, this review will motivate researchers from diverse disciplinary fields to explore the potential applications of HG and its derivatives more deeply.

## Figures and Tables

**Table:** 

**Aβ**	Amyloid β
**AChE**	Acetylcholinesterase
**AD**	Alzheimer’s disease
**AKI**	Acute kidney injury
**AKT**	Protein kinase B
**ALI**	Acute lung injury
**ALP**	Alkaline phosphatase
**ALT**	Alanine aminotransferase
**AMPK**	Adenosine-monophosphate-activated protein kinase
**ARE**	Antioxidant response element
**AST**	Aspartate aminotransferase
**Axin2**	Axis inhibition protein 2
**AZT**	Zidovudine
**11β-HSD1**	11β-hydroxysteroid dehydrogenase type 1
**CKD**	Chronic kidney disease
**Cmax**	Peak concentration
**COL Iα1**	Collagen type I α 1 chain
**CORT**	Corticosterone
**COX-2**	Cyclooxygenase-2
**CYP**	Cytochrome P450
**CYP51**	Sterol 14a-demethylase
**Cyt c**	Cytochrome c
**DA**	Dopamine
**DM**	Diabetes mellitus
**DPCK**	Dephospho-coenzyme A-kinase
**Drp1**	Dynamin-related protein 1
**EC**_**50**_	Half-maximal effective concentration
***E. faecalis***	*Enterococcus faecalis*
**EGFR**	Epidermal growth factor receptor
**EMT**	Epithelial-mesenchymal transition
**ERK**	Extracellular signal-regulated kinase
**FAE**	*Fructus Akebiae* extracts
**FAS**	Fatty acid synthase
***F. hepatica***	*Fasciola hepatica*
**FXR**	Farnesoid X receptor
**GP**	Glycogen phosphorylase
**GSH**	Glutathione
**HDL**	High-density lipoprotein
**HG**	Hederagenin
**HNC**	Head and neck cancer
**HO-1**	Heme oxygenase-1
**HPA**	Hypothalamic-pituitary-adrenal
**HPC**	Hepatic parenchymal cell
**HSC**	Hepatic stellate cell
**5-HT**	Serotonin
**IC**_**50**_	Half-maximal inhibitory concentration
**IFN**	Interferon
**IκBα**	Inhibitor of kappa Bα
**IKK**	Inhibitory-kappa B kinase
**IL**	Interleukin
**iNOS**	inducible Nitric oxide synthase
**IRF3**	Interferon regulatory factor 3
**ISG15**	Interferon-stimulated gene15
**JAK2**	Janus kinase 2
**JNK**	c-Jun NH2-terminal kinase
**Keap1**	Kelch-like ECH-associated protein 1
**KIF7**	Kinesin family member 7
**LC3**	Light chain 3
**LDL**	Low-density lipoprotein
***L. infantum***	*Leishmania infantum*
**LncRNA**	Long noncoding RNA
**LncRNA A33**	LncRNA A330074k22Rik
**LOX**	Lipoxygenase
**LPS**	Lipopolysaccharide
**M3 receptor**	M3 muscarinic acetylcholine receptor
**MAPK**	Mitogen-activated protein kinase
**MDA**	Malondialdehyde
**MDR**	Multidrug resistance
**MIC**	Minimum inhibitory concentration
**miRNA**	microRNA
**MLK3**	Mixed lineage kinase 3
**MMP**	Mitochondrial membrane potential
**mTOR**	mammalian Target of rapamycin
**NE**	Norepinephrine
**NFAT4**	Nuclear factor of activated T cells 4
**NF-κB**	Nuclear factor-kappa B
**NLRP3**	NOD-like receptor pyrin domain-containing 3
**NO**	Nitric oxide
**Nrf2**	Nuclear factor erythroid 2-related factor 2
**NSCLC**	Non-small cell lung cancer
**PARP**	Poly ADP-ribose polymerase
**PEG**	Polyethylene glycol
**PGE**_**2**_	Prostaglandin E_2_
**P-gp**	P-glycoprotein
**PI3K**	Phosphatidylinositol 3 kinase
**PLY**	Pneumolysin
**PPARα**	Peroxisome proliferator-activated receptor α
**PTPN1**	Protein tyrosine phosphatase nonreceptor type 1
**ROS**	Reactive oxygen species
**RUNX2**	Runt-related transcription factor 2
**SMA**	Smooth muscle actin
**SOD**	Superoxide dismutase
***S. pneumoniae***	*Streptococcus pneumoniae*
**STAT**	Signal transducer and activator of transcription
**STING**	Stimulator of interferon genes
**T1/2**	Half-life
**TBK1**	TANK-binding kinase 1
**TC**	Total cholesterol
**TFEB**	Transcription factor EB
**TG**	Triglyceride
**TGF**	Transforming growth factor
***T. gondii***	*Toxoplasma gondii*
**Tmax**	Time to reach the maximum concentration
**TNF**	Tumor necrosis factor

## References

[B1] Alcorn J. F. van der Velden J. Brown A. L. McElhinney B. Irvin C. G. Janssen-Heininger Y. M. (2009). c-Jun N-terminal kinase 1 is required for the development of pulmonary fibrosis. Am. J. Respir. Cell Mol. Biol. 40 (4), 422–432. 10.1165/rcmb.2008-0174OC 18836136 PMC2660560

[B2] Amaravadi R. K. Kimmelman A. C. Debnath J. (2019). Targeting autophagy in cancer: recent advances and future directions. Cancer Discov. 9 (9), 1167–1181. 10.1158/2159-8290.Cd-19-0292 31434711 PMC7306856

[B3] Anderson O. Beckett J. Briggs C. C. Natrass L. A. Cranston C. F. Wilkinson E. J. (2020). An investigation of the antileishmanial properties of semi-synthetic saponins. RSC Med. Chem. 11 (7), 833–842. 10.1039/d0md00123f 33479679 PMC7651632

[B4] Baek S. J. Lee H. Park S. M. Park M. Yi J. M. Kim N. S. (2022). Identification of a novel anticancer mechanism of Paeoniae Radix extracts based on systematic transcriptome analysis. Biomed. Pharmacother. 148, 112748. 10.1016/j.biopha.2022.112748 35219117

[B5] Bai H. Yang B. Yu W. Xiao Y. Yu D. Zhang Q. (2018). Cathepsin B links oxidative stress to the activation of NLRP3 inflammasome. Exp. Cell Res. 362 (1), 180–187. 10.1016/j.yexcr.2017.11.015 29196167

[B6] Balka K. R. Louis C. Saunders T. L. Smith A. M. Calleja D. J. D'Silva D. B. (2020). TBK1 and IKKε act redundantly to mediate STING-induced NF-κB responses in myeloid cells. Cell Rep. 31, 107492. 10.1016/j.celrep.2020.03.056 32268090

[B7] Burris H. A. (2013). Overcoming acquired resistance to anticancer therapy: focus on the PI3K/AKT/mTOR pathway. Cancer Chemother. Pharmacol. 71 (4), 829–842. 10.1007/s00280-012-2043-3 23377372

[B8] Callaway D. A. Jiang J. X. (2015). Reactive oxygen species and oxidative stress in osteoclastogenesis, skeletal aging and bone diseases. J. Bone Min. Metab. 33 (4), 359–370. 10.1007/s00774-015-0656-4 25804315

[B9] Chakroborty A. Pritchard D. R. Bouillon M. E. Cervi A. Kraehenbuehl R. Wild C. (2023). Modified hederagenin derivatives demonstrate *ex vivo* anthelmintic activity against Fasciola hepatica. Pharmaceutics 15 (7), 1869. 10.3390/pharmaceutics15071869 37514055 PMC10385850

[B10] Chen B. Ning K. Sun M. L. Zhang X. A. (2023). Regulation and therapy, the role of JAK2/STAT3 signaling pathway in OA: a systematic review. Cell Commun. Signal 21 (1), 67. 10.1186/s12964-023-01094-4 37013568 PMC10071628

[B11] Chen B. Zhang Q. Wang W. Huang H. Kang W. (2012). Alpha-glucosidase inhibitory active constituents contained in nutshell of Trapa acornis. China J. Chin. Mater Med. 37 (10), 1408–1411. 10.4268/cjcmm20121013 22860451

[B12] Chen Z. Huang K. Y. Ling Y. Goto M. Duan H. Q. Tong X. H. (2019). Discovery of an oleanolic acid/hederagenin-nitric oxide donor hybrid as an EGFR tyrosine kinase inhibitor for non-small-cell lung cancer. J. Nat. Prod. 82 (11), 3065–3073. 10.1021/acs.jnatprod.9b00659 31718182

[B13] Chen Z. K. Zhao D. Feng S. X. Xu J. (2022). Pharmacodynamics and cellular uptake of peimine and peiminine in inflammatory model non-small-cell lung cancer epithelial cells (A549). Evid. Based Complement. Altern. Med. 2022, 2946201. 10.1155/2022/2946201 PMC8843782 35178100

[B14] Chow C. W. Rincón M. Cavanagh J. Dickens M. Davis R. J. (1997). Nuclear accumulation of NFAT4 opposed by the JNK signal transduction pathway. Science 278 (5343), 1638–1641. 10.1126/science.278.5343.1638 9374467

[B15] Chowdhury M. A. Ko H. J. Lee H. Aminul Haque M. Park I. S. Lee D. S. (2017). Oleanane triterpenoids from Akebiae Caulis exhibit inhibitory effects on Aβ42 induced fibrillogenesis. Arch. Pharm. Res. 40 (3), 318–327. 10.1007/s12272-016-0885-7 28054176

[B16] Cotton J. A. Durrant C. Franssen S. U. Gelanew T. Hailu A. Mateus D. (2020). Genomic analysis of natural intra-specific hybrids among Ethiopian isolates of Leishmania donovani. PLoS Negl. Trop. Dis. 14 (4), e0007143. 10.1371/journal.pntd.0007143 32310945 PMC7237039

[B17] Cwiklinski K. O'Neill S. M. Donnelly S. Dalton J. P. (2016). A prospective view of animal and human Fasciolosis. Parasite Immunol. 38 (9), 558–568. 10.1111/pim.12343 27314903 PMC5053257

[B18] Dai Y. Masra N. Zhou L. Yu C. Jin W. Ni H. (2023). Hederagenin suppresses glioma cell biological activities via Nur77 *in vitro* study. Food Sci. Nutr. 11 (3), 1283–1296. 10.1002/fsn3.3163 36911825 PMC10002964

[B19] Ding R. Zhang Y. Xu X. Hou Y. Nie J. Deng X. (2022). Inhibitory effect of hederagenin on Streptococcus pneumoniae pneumolysin *in vitro* . Microbes Infect. 24 (2), 104888. 10.1016/j.micinf.2021.104888 34547436

[B20] Ding Y. Li W. Peng S. Zhou G. Chen S. Wei Y. (2023). Puerarin protects against myocardial ischemia/reperfusion injury by inhibiting ferroptosis. Biol. Pharm. Bull. 46 (4), 524–532. 10.1248/bpb.b22-00174 36696989

[B21] Eastell R. O'Neill T. W. Hofbauer L. C. Langdahl B. Reid I. R. Gold D. T. (2016). Postmenopausal osteoporosis. Nat. Rev. Dis. Prim. 2, 16069. 10.1038/nrdp.2016.69 27681935

[B22] Fallon C. M. Smyth J. S. Quach A. Lajczak-McGinley N. O'Toole A. Barrett K. E. (2022). Pentacyclic triterpenes modulate farnesoid X receptor expression in colonic epithelial cells: implications for colonic secretory function. J. Biol. Chem. 298 (11), 102569. 10.1016/j.jbc.2022.102569 36209824 PMC9663526

[B23] Fang K. Zhang X. H. Han Y. T. Wu G. R. Cai D. S. Xue N. N. (2018). Design, synthesis, and cytotoxic analysis of novel Hederagenin⁻Pyrazine derivatives based on partial least squares discriminant analysis. Int. J. Mol. Sci. 19 (10), 2994. 10.3390/ijms19102994 30274380 PMC6213900

[B24] Fang L. Liu M. Cai L. (2019). Hederagenin inhibits proliferation and promotes apoptosis of cervical cancer CaSki cells by blocking STAT3 pathway. Xi Bao Yu Fen Zi Mian Yi Xue Za Zhi 35 (2), 140–145. 10.13423/j.cnki.cjcmi.008775 30975278

[B25] Fernandes V. E. Ercoli G. Bénard A. Brandl C. Fahnenstiel H. Müller-Winkler J. (2020). The B-cell inhibitory receptor CD22 is a major factor in host resistance to Streptococcus pneumoniae infection. PLoS Pathog. 16 (4), e1008464. 10.1371/journal.ppat.1008464 32324805 PMC7179836

[B26] Gao Y. He C. Bi W. Wu G. Altman E. (2016). Bioassay guided fractionation identified hederagenin as a major cytotoxic agent from Cyclocarya paliurus leaves. Planta Med. 82 (1-2), 171–179. 10.1055/s-0035-1557900 26393939

[B27] Gauthier C. Legault J. Girard-Lalancette K. Mshvildadze V. Pichette A. (2009). Haemolytic activity, cytotoxicity and membrane cell permeabilization of semi-synthetic and natural lupane- and oleanane-type saponins. Bioorg. Med. Chem. 17 (5), 2002–2008. 10.1016/j.bmc.2009.01.022 19200744

[B28] Ge W. Chen J. W. Li X. Li J. Z. Cai H. D. Tao Q. (2023). Chemical constituents from aerial parts of Saururus chinensis. Chin. Tradit. Herb. Drugs 54 (11), 3417–3423. 10.7501/j.issn.0253-2670.2023.11.003

[B29] Grosse M. Günther K. Jordan P. M. Roman D. Werz O. Beemelmanns C. (2022). Synthesis of functionalized δ-Hydroxy-β-keto esters and evaluation of their anti-inflammatory properties. Chembiochem 23, e202200073. 10.1002/cbic.202200073 35244320 PMC9314795

[B30] Guan Y. Z. Liu H. Huang H. J. Liang D. Y. Wu S. Y. Zhang T. (2022). Identification of the potential molecular mechanism of TGFBI gene in persistent atrial fibrillation. Comput. Math. Methods Med. 2022, 1643674. 10.1155/2022/1643674 36398072 PMC9666036

[B31] Gudkova D. Panasyuk G. Nemazanyy I. Zhyvoloup A. Monteil P. Filonenko V. (2012). EDC4 interacts with and regulates the dephospho-CoA kinase activity of CoA synthase. FEBS Lett. 586 (20), 3590–3595. 10.1016/j.febslet.2012.08.033 22982864

[B32] Haam J. Yakel J. L. (2017). Cholinergic modulation of the hippocampal region and memory function. J. Neurochem. 142 (Suppl. 2), 111–121. 10.1111/jnc.14052 28791706 PMC5645066

[B33] Han Z. Ren W. Liu X. Lin N. Qu J. Duan X. (2024). Hypoglycemic activity of immature persimmon (Diospyros kaki Thunb.) extracts and its inhibition mechanism for α-amylase and α-glucosidase. Int. J. Biol. Macromol. 257 (Pt 2), 128616. 10.1016/j.ijbiomac.2023.128616 38070815

[B34] Hao Y. J. Wei Y. Zhou Y. Liu Q. Shen F. Xiang Q. (2021). Conjugates of hederagenin hemiester derivatives with AZT as virus protease inhibitors. Chin. J. Syn. Chem. 29 (9). 10.15952/j.cnki.cjsc.1005-1511.21129

[B35] Haupt M. Gerner S. T. Bähr M. Doeppner T. R. (2023). Neuroprotective strategies for ischemic stroke-future perspectives. Int. J. Mol. Sci. 24 (5), 4334. 10.3390/ijms24054334 36901765 PMC10002358

[B36] Hill D. E. Chirukandoth S. Dubey J. P. (2005). Biology and epidemiology of Toxoplasma gondii in man and animals. Anim. Health Res. Rev. 6 (1), 41–61. 10.1079/ahr2005100 16164008

[B37] Hong K. W. Jia X. S. Yang X. J. Dong D. X. (2017). Synthesis and *in vitro* anti-HBV activities of novel hederagenin derivatives. Chin. J. Synthetic Chem. 25 (1), 18–25. 10.15952/j.cnki.cjsc.1005-1511.2017.01.16250

[B38] Hu L. Zhang Y. Miao W. Cheng T. (2019). Reactive oxygen species and Nrf2: functional and transcriptional regulators of hematopoiesis. Oxid. Med. Cell Longev. 2019, 5153268. 10.1155/2019/5153268 31827678 PMC6885799

[B39] Hu S. Q. Ye J. S. Zong Y. Y. Sun C. C. Liu D. H. Wu Y. P. (2012). S-nitrosylation of mixed lineage kinase 3 contributes to its activation after cerebral ischemia. J. Biol. Chem. 287 (4), 2364–2377. 10.1074/jbc.M111.227124 22123824 PMC3268398

[B40] Huai Y. Zhang W. J. Wang W. Dang K. Jiang S. F. Li D. M. (2021). Systems pharmacology dissection of action mechanisms for herbs in osteoporosis treatment. Chin. Herb. Med. 13 (3), 313–331. 10.1016/j.chmed.2021.06.001 36118922 PMC9476722

[B41] Huang J. Chen R. Ban Y. J. Liu W. X. Zhu G. F. Fang Y. (2022a). Inhibitory effects of hederagenin on five common cytochrome P450 enzymes in human liver microsomes *in vitro* . Chin. J. Clin. Pharmacol. 38 (9), 993–997. 10.13699/j.cnki.1001-6821.2022.09.022

[B42] Huang L. Li Q. (2020). Notoginsenoside R1 promotes differentiation of human alveolar osteoblasts in inflammatory microenvironment through inhibiting NF‑κB pathway and activating Wnt/β‑catenin pathway. Mol. Med. Rep. 22 (6), 4754–4762. 10.3892/mmr.2020.11537 33174026 PMC7646889

[B43] Huang W. Wang Y. Xu S. Qiao H. Cheng H. Wang L. (2022b). Design, synthesis, and tumor drug resistance reversal activity of novel hederagenin derivatives modified by nitrogen-containing heterocycles. Eur. J. Med. Chem. 232, 114207. 10.1016/j.ejmech.2022.114207 35219948

[B44] Huang X. Shen Q. K. Guo H. Y. Li X. Quan Z. S. (2023). Pharmacological overview of hederagenin and its derivatives. RSC Med. Chem. 14 (10), 1858–1884. 10.1039/d3md00296a 37859723 PMC10583830

[B45] Huang Z. Fu J. Zhang Y. (2017). Nitric oxide donor-based cancer therapy: advances and prospects. J. Med. Chem. 60 (18), 7617–7635. 10.1021/acs.jmedchem.6b01672 28505442

[B46] Inoue-Yamauchi A. Oda H. (2012). Depletion of mitochondrial fission factor DRP1 causes increased apoptosis in human colon cancer cells. Biochem. Biophys. Res. Commun. 421 (1), 81–85. 10.1016/j.bbrc.2012.03.118 22487795

[B47] Jia J. Xu L. H. Deng C. Zhong X. Xie K. H. Han R. Y. (2023). Hederagenin ameliorates renal fibrosis in chronic kidney disease through blocking ISG15 regulated JAK/STAT signaling. Int. Immunopharmacol. 118, 110122. 10.1016/j.intimp.2023.110122 37023701

[B48] Jiang Y. Zou D. Li Y. Gu S. Dong J. Ma X. (2022). Monoamine neurotransmitters control basic emotions and affect major depressive disorders. Pharm. (Basel) 15 (10), 1203. 10.3390/ph15101203 PMC9611768 36297314

[B49] Jin J. L. Lee Y. Y. Heo J. E. Lee S. Kim J. M. Yun-Choi H. S. (2004). Anti-platelet pentacyclic triterpenoids from leaves of Campsis grandiflora. Arch. Pharm. Res. 27 (4), 376–380. 10.1007/bf02980076 15180300

[B50] Jin Z. L. Gao N. Zhou D. Chi M. G. Yang X. M. Xu J. P. (2012). The extracts of Fructus Akebiae, a preparation containing 90% of the active ingredient hederagenin: serotonin, norepinephrine and dopamine reuptake inhibitor. Pharmacol. Biochem. Behav. 100 (3), 431–439. 10.1016/j.pbb.2011.10.001 22005599

[B51] Kanda A. Kawai H. Suto S. Kitajima S. Sato S. Takata T. (2005). Aurora-B/AIM-1 kinase activity is involved in Ras-mediated cell transformation. Oncogene 24 (49), 7266–7272. 10.1038/sj.onc.1208884 16027732

[B52] Kayangar M. Ngansop Nono R. Kühlborn J. Tchuenguem R. Ponou B. K. Jenett-Siems K. (2019). A new ursane-type triterpene oxoglucopyranoside from Crossopteryx febrifuga. Z. Naturforsch. C J. Biosci. 74 (11-12), 289–293. 10.1515/znc-2019-0113 31525160

[B53] Kim E. H. Baek S. Shin D. Lee J. Roh J. L. (2017a). Hederagenin induces apoptosis in cisplatin-resistant head and neck cancer cells by inhibiting the nrf2-ARE antioxidant pathway. Oxid. Med. Cell. Longev. 2017, 5498908. 10.1155/2017/5498908 29456786 PMC5804377

[B54] Kim G. J. Song D. H. Yoo H. S. Chung K. H. Lee K. J. An J. H. (2017b). Hederagenin supplementation alleviates the pro-inflammatory and apoptotic response to alcohol in rats. Nutrients 9, 41. 10.3390/nu9010041 28067819 PMC5295085

[B55] Kim S. Y. Son K. H. Chang H. W. Kang S. S. Kim H. P. (1999). Inhibition of mouse ear edema by steroidal and triterpenoid saponins. Arch. Pharm. Res. 22, 313–316. 10.1007/bf02976370 10403138

[B56] Komori T. (2010). Regulation of bone development and extracellular matrix protein genes by RUNX2. Cell Tissue Res. 339 (1), 189–195. 10.1007/s00441-009-0832-8 19649655

[B57] Kondo Y. Kanzawa T. Sawaya R. Kondo S. (2005). The role of autophagy in cancer development and response to therapy. Nat. Rev. Cancer 5 (9), 726–734. 10.1038/nrc1692 16148885

[B58] Lee C. W. Park S. M. Zhao R. Lee C. Chun W. Son Y. (2015). Hederagenin, a major component of Clematis mandshurica Ruprecht root, attenuates inflammatory responses in RAW 264.7 cells and in mice. Int. Immunopharmacol. 29 (2), 528–537. 10.1016/j.intimp.2015.10.002 26481049

[B59] Lee K. J. Ratih K. Kim G. J. Lee Y. R. Shin J. S. Chung K. H. (2022). Immunomodulatory and anti-inflammatory efficacy of hederagenin-coated maghemite (γ-Fe2O3) nanoparticles in an atopic dermatitis model. Colloids Surf. B Biointerfaces 210, 112244. 10.1016/j.colsurfb.2021.112244 34896691

[B60] Levy J. M. M. Towers C. G. Thorburn A. (2017). Targeting autophagy in cancer. Nat. Rev. Cancer 17 (9), 528–542. 10.1038/nrc.2017.53 28751651 PMC5975367

[B61] Li C. T. Xie H. Zhang D. Song Y. X. (2013a). Determination of hederagenin in root of Luffa cylindrica. Chin. J. Exp. Tradit. Med. Formulae 19 (18), 145–147. 10.11653/syfj2013180145

[B62] Li H. Xie W. Gao X. Geng Z. Gao J. Ma G. (2024a). Design and synthesis of novel hederagonic acid analogs as potent anti-inflammatory compounds capable of protecting against LPS-induced acute lung injury. Eur. J. Med. Chem. 263, 115941. 10.1016/j.ejmech.2023.115941 38000214

[B63] Li J. F. Chen J. F. Tang S. L. Ma R. Q. (2013b). Determination of hederagenin in Radix Dipsaci from different habitats by HPLC. Tradit. Chin. Drug Res. Clin. Pharmacol. 24 (6), 606–609. 10.3969/j.issn.1003-9783.2013.06.020

[B64] Li K. Li S. Wang D. Li X. Wu X. Liu X. (2019a). Extraction, characterization, antitumor and immunological activities of hemicellulose polysaccharide from Astragalus radix herb residue. Molecules 24 (20), 3644. 10.3390/molecules24203644 31601012 PMC6833037

[B65] Li K. Wang Y. Ni H. (2023). Hederagenin upregulates PTPN1 expression in Aβ-stimulated neuronal cells, exerting anti-oxidative stress and anti-apoptotic activities. J. Mol. Neurosci. 73 (11-12), 932–945. 10.1007/s12031-023-02160-9 37882913

[B66] Li Q. Q. Ma C. H. Liu C. S. Xiao Y. Chen M. L. Tian Z. H. (2013c). Quality survey of different species of Clematidis Radix et Rhizoma. China J. Chin. Mater Med. 38 (8), 1203–1205. 10.4268/cjcmm20130817 23944036

[B67] Li X. Wang Y. Li L. Zhou S. Zhao F. (2021). Sclareol inhibits RANKL-induced osteoclastogenesis and promotes osteoblastogenesis through promoting CCN1 expression via repressing the MAPK pathway. Cell Biol. Toxicol. 37 (6), 849–871. 10.1007/s10565-020-09578-6 33423118

[B68] Li Y. Dong J. Shang Y. Zhao Q. Li P. Wu B. (2019b). Anti-inflammatory effects of hederagenin on diabetic cardiomyopathy via inhibiting NF-κB and Smads signaling pathways in a type-2 diabetic mice model. RSC Adv. 9 (45), 26238–26247. 10.1039/c9ra02043h 35531007 PMC9070383

[B69] Li Y. H. Lu Z. L. Wei J. C. Huang R. S. Lei P. L. Pan Z. Z. (2020). Establishment a method for the content determination of hederagenin in Hedera Sinensis Hebra. J. Liaoning Univ. Tradit. Chin. Med. 22 (1), 63–66. 10.13194/j.issn.1673-842x.2020.01.018

[B70] Li Y. Y. Qin Z. H. Sheng R. (2024b). The multiple roles of autophagy in neural function and diseases. Neurosci. Bull. 40 (3), 363–382. 10.1007/s12264-023-01120-y 37856037 PMC10912456

[B71] Lian J. Hua T. Xu J. Ding J. Liu Z. Fan Y. (2021). Interleukin-1β weakens paclitaxel sensitivity through regulating autophagy in the non-small cell lung cancer cell line A549. Exp. Ther. Med. 21 (4), 293. 10.3892/etm.2021.9724 33717236 PMC7885084

[B72] Liang B. F. Huang F. Wang H. T. Wang G. H. Yuan X. Zhang M. Z. (2015). Involvement of norepinephrine and serotonin system in antidepressant-like effects of hederagenin in the rat model of unpredictable chronic mild stress-induced depression. Pharm. Biol. 53 (3), 368–377. 10.3109/13880209.2014.922586 25471378

[B73] Liang P. Jiang B. Li Y. Liu Z. Zhang P. Zhang M. (2018). Autophagy promotes angiogenesis via AMPK/Akt/mTOR signaling during the recovery of heat-denatured endothelial cells. Cell Death Dis. 9 (12), 1152. 10.1038/s41419-018-1194-5 30455420 PMC6242874

[B74] Liang W. Xie Z. Liao D. Li Y. Li Z. Zhao Y. (2023). Inhibiting microRNA-142-5p improves learning and memory in Alzheimer's disease rats via targeted regulation of the PTPN1-mediated Akt pathway. Brain Res. Bull. 192, 107–114. 10.1016/j.brainresbull.2022.02.016 35219754

[B75] Lin R. Liu L. Silva M. Fang J. Zhou Z. Wang H. (2021). Hederagenin protects PC12 cells against corticosterone-induced injury by the activation of the PI3K/AKT pathway. Front. Pharmacol. 12, 712876. 10.3389/fphar.2021.712876 34721013 PMC8551867

[B76] Liu B. Su H. (2023). Luteolin improves vasoconstriction function and survival of septic mice via AMPK/NF-κB pathway. Heliyon 9, e13330. 10.1016/j.heliyon.2023.e13330 36816271 PMC9932738

[B77] Liu B. X. Zhou J. Y. Li Y. Zou X. Wu J. Gu J. F. (2014a). Hederagenin from the leaves of ivy (Hedera helix L.) induces apoptosis in human LoVo colon cells through the mitochondrial pathway. BMC Complement. Altern. Med. 14, 412. 10.1186/1472-6882-14-412 25342273 PMC4216349

[B78] Liu E. W. Wang J. L. Han L. F. Chang Y. X. Wang T. Huo Y. (2014b). Pharmacokinetics study of asperosaponin VI and its metabolites cauloside A, HN saponin F and hederagenin. J. Nat. Med. 68 (3), 488–497. 10.1007/s11418-014-0821-4 24615060

[B79] Liu J. M. Gao Y. H. Xu H. H. Xu Z. Q. (2007). Chemical constituents of Lignum Aquilariae Resinatum. Chin. Tradit. Herb. Drugs 38 (8).

[B80] Liu X. Gao S. Xu H. (2018). lncRNAPCAT29 inhibits pulmonary fibrosis via the TGF‑β1‑regulated RASAL1/ERK1/2 signal pathway. Mol. Med. Rep. 17 (6), 7781–7788. 10.3892/mmr.2018.8807 29620190

[B81] Liu X. Sun L. Liu Q. H. Chen B. Q. Liu Y. M. (2020a). Synthesis, characterization and anti-hepatoma activity of new hederagenin derivatives. Mini Rev. Med. Chem. 20 (3), 252–257. 10.2174/1389557519666191010091612 32134368

[B82] Liu X. X. Yang Y. T. Wang X. Wang K. Y. Liu J. Q. Lei L. (2017). Design, synthesis and biological evaluation of novel α-hederagenin derivatives with anticancer activity. Eur. J. Med. Chem. 141, 427–439. 10.1016/j.ejmech.2017.09.016 29040953

[B83] Liu Y. Feng Q. Miao J. Wu Q. Zhou S. Shen W. (2020b). C-X-C motif chemokine receptor 4 aggravates renal fibrosis through activating JAK/STAT/GSK3β/β-catenin pathway. J. Cell Mol. Med. 24 (7), 3837–3855. 10.1111/jcmm.14973 32119183 PMC7171406

[B84] Loftus T. M. Jaworsky D. E. Frehywot G. L. Townsend C. A. Ronnett G. V. Lane M. D. (2000). Reduced food intake and body weight in mice treated with fatty acid synthase inhibitors. Science 288 (5475), 2379–2381. 10.1126/science.288.5475.2379 10875926

[B85] Lu S. H. Guan J. H. Huang Y. L. Pan Y. W. Yang W. Lan H. (2015). Experimental study of antiatherosclerosis effects with hederagenin in rats. Evid. Based Complement. Altern. Med. 2015, 456354. 10.1155/2015/456354 PMC4629025 26557859

[B86] Lu Y. L. Yu L. Zhu T. T. Zhang N. Chen L. L. Hang T. J. (2012). Determination of hederagenin and oleanolic acid in Schefflera arboricola. Chin. J. Pharm. Anal. 32 (11), 1945–1949. 10.16155/j.0254-1793.2012.11.007

[B87] Luo D. Z. Fu R. R. Gan Y. F. Wang M. Q. Chen H. Y. Huang H. L. (2020a). Chemical constituents of Syzygium grijsii. J. Chin. Med. Mater 43 (5), 1130–1133. 10.13863/j.issn1001-4454.2020.05.017

[B88] Luo J. G. Liu J. Kong L. Y. (2008). New pentacyclic triterpenes from Gypsophila oldhamiana and their biological evaluation as glycogen phosphorylase inhibitors. Chem. Biodivers. 5 (5), 751–757. 10.1002/cbdv.200890071 18493961

[B89] Luo R. Su L. Y. Li G. Yang J. Liu Q. Yang L. X. (2020b). Activation of PPARA-mediated autophagy reduces Alzheimer disease-like pathology and cognitive decline in a murine model. Autophagy 16 (1), 52–69. 10.1080/15548627.2019.1596488 30898012 PMC6984507

[B90] Ma R. Q. Fang C. Zhang Y. Wu P. N. Qiu Y. W. (2021). Comparative pharmacokinetic study of hederagenin and its derivatives in beagle dogs. J. Chin. Med. Mater. 44 (1), 200–204. 10.13863/j.issn1001-4454.2021.01.038

[B211] Ma T. F. Fan Y. R. Zhao Y. H. Liu B. (2023). Emerging role of autophagy in colorectal cancer: progress and prospects for clinical intervention. World J. Gastrointest. Oncol. 15 (6), 979–987. 10.4251/wjgo.v15.i6.979 37389106 PMC10302997

[B91] Ma W. Huang Q. Xiong G. Deng L. He Y. (2020). The protective effect of Hederagenin on pulmonary fibrosis by regulating the Ras/JNK/NFAT4 axis in rats. Biosci. Biotechnol. Biochem. 84 (6), 1131–1138. 10.1080/09168451.2020.1721263 32024440

[B92] Ma W. Z. Ling T. J. Zhang Y. H. Lin L. D. (2005). Chemical constituents from Nauclea officinalis. J. Trop. Subtrop. Bot. 13 (2), 167–170.

[B93] Maiuri M. C. Zalckvar E. Kimchi A. Kroemer G. (2007). Self-eating and self-killing: crosstalk between autophagy and apoptosis. Nat. Rev. Mol. Cell Biol. 8 (9), 741–752. 10.1038/nrm2239 17717517

[B94] Majester-Savornin B. Elias R. Diaz-Lanza A. M. Balansard G. Gasquet M. Delmas F. (1991). Saponins of the ivy plant, Hedera helix, and their leishmanicidic activity. Planta Med. 57 (3), 260–262. 10.1055/s-2006-960086 1896525

[B95] Masenga S. K. Kabwe L. S. Chakulya M. Kirabo A. (2023). Mechanisms of oxidative stress in metabolic syndrome. Int. J. Mol. Sci. 24 (9), 7898. 10.3390/ijms24097898 37175603 PMC10178199

[B96] McCall L. I. El Aroussi A. Choi J. Y. Vieira D. F. De Muylder G. Johnston J. B. (2015). Targeting Ergosterol biosynthesis in Leishmania donovani: essentiality of sterol 14 alpha-demethylase. PLoS Negl. Trop. Dis. 9 (3), e0003588. 10.1371/journal.pntd.0003588 25768284 PMC4359151

[B97] Menpadi N. Prakash J. Kundu D. Chandra P. Dubey V. K. (2023). Integrated computational and experimental approach for novel anti-leishmanial molecules by targeting Dephospho-coenzyme A kinase. Int. J. Biol. Macromol. 232, 123441. 10.1016/j.ijbiomac.2023.123441 36708902

[B98] Mfotie Njoya E. Ndemangou B. Akinyelu J. Munvera A. M. Chukwuma C. I. Mkounga P. (2023). *In vitro* antiproliferative, anti-inflammatory effects and molecular docking studies of natural compounds isolated from Sarcocephalus pobeguinii (Hua ex Pobég). Front. Pharmacol. 14, 1205414. 10.3389/fphar.2023.1205414 37416061 PMC10320002

[B99] Monisha J. Roy N. K. Bordoloi D. Kumar A. Golla R. Kotoky J. (2017). Nuclear factor kappa B: a potential target to persecute head and neck cancer. Curr. Drug Targets 18 (2), 232–253. 10.2174/1389450117666160201112330 26844566

[B100] Mostofi F. Mostofi M. Niroomand B. Hosseini S. Alipour A. Homaeigohar S. (2022). Crossing phylums: butterfly wing as a natural perfusable three-dimensional (3D) bioconstruct for bone tissue engineering. J. Funct. Biomater. 13 (2), 68. 10.3390/jfb13020068 35735923 PMC9225241

[B101] Nakka V. P. Mohammed A. Q. (2020). A critical role for ISGylation, ubiquitination and, SUMOylation in brain damage: implications for neuroprotection. Neurochem. Res. 45 (9), 1975–1985. 10.1007/s11064-020-03066-3 32500407

[B102] Nilsson M. B. Sun H. Robichaux J. Pfeifer M. McDermott U. Travers J. (2020). A YAP/FOXM1 axis mediates EMT-associated EGFR inhibitor resistance and increased expression of spindle assembly checkpoint components. Sci. Transl. Med. 12 (559), eaaz4589. 10.1126/scitranslmed.aaz4589 32878980 PMC8269000

[B103] Oh S. R. Jung K. Y. Son K. H. Park S. H. Lee I. S. Ahn K. S. (1999). *In vitro* anticomplementary activity of hederagenin saponins isolated from roots of Dipsacus asper. Arch. Pharm. Res. 22 (3), 317–319. 10.1007/bf02976371 10403139

[B104] Oladimeji A. O. Oladosu I. A. Jabeen A. Faheem A. Mesaik M. A. Ali M. S. (2017). Immunomodulatory activities of isolated compounds from the root-bark of Cussonia arborea. Pharm. Biol. 55 (1), 2240–2247. 10.1080/13880209.2017.1400078 29141487 PMC6130543

[B105] Ou Y. B. Wei M. J. Li H. R. (2013). Chemical constituents of Quercus pannosa. Chin. Tradit. Herb. Drugs 44 (14), 1872–1876. 10.7501/j.issn.0253-2670.2013.14.002

[B106] Pak E. Segal R. A. (2016). Hedgehog signal transduction: key players, oncogenic drivers, and cancer therapy. Dev. Cell 38 (4), 333–344. 10.1016/j.devcel.2016.07.026 27554855 PMC5017307

[B107] Pan X. Chen T. Zhang Z. Chen X. Chen C. Chen L. (2019). Activation of Nrf2/HO-1 signal with Myricetin for attenuating ECM degradation in human chondrocytes and ameliorating the murine osteoarthritis. Int. Immunopharmacol. 75, 105742. 10.1016/j.intimp.2019.105742 31325727

[B108] Park H. J. Kim D. H. Choi J. W. Park J. H. Han Y. N. (1998). A potent anti-diabetic agent from Kalopanax pictus. Arch. Pharm. Res. 21 (1), 24–29. 10.1007/bf03216748 9875510

[B109] Picciotto M. R. Higley M. J. Mineur Y. S. (2012). Acetylcholine as a neuromodulator: cholinergic signaling shapes nervous system function and behavior. Neuron 76 (1), 116–129. 10.1016/j.neuron.2012.08.036 23040810 PMC3466476

[B110] Qian Z. M. Li H. J. Li P. He Q. H. Qi F. F. (2006). Triterpenoids from the aerial parts of Lonicera syringantha Maxim. Chem Ind For Prod 24 (4), 23–25.

[B111] Reed K. M. Mendoza K. M. Coulombe R. A. Jr. (2007). Structure and genetic mapping of the Cytochrome P450 gene (CYP1A5) in the Turkey (*Meleagris gallopavo*). Cytogenet. Genome Res. 116 (1-2), 104–109. 10.1159/000097426 17268186

[B112] Ren Y. Li L. Wan L. Huang Y. Cao S. (2022). Glucokinase as an emerging anti-diabetes target and recent progress in the development of its agonists. J. Enzyme Inhib. Med. Chem. 37 (1), 606–615. 10.1080/14756366.2021.2025362 35067153 PMC8788356

[B113] Rezatabar S. Karimian A. Rameshknia V. Parsian H. Majidinia M. Kopi T. A. (2019). RAS/MAPK signaling functions in oxidative stress, DNA damage response and cancer progression. J. Cell Physiol. 234 (9), 14951–14965. 10.1002/jcp.28334 30811039

[B114] Rodríguez-Hernández D. Barbosa L. C. A. Demuner A. J. Ataide Martins J. P. Fischer Nee Heller L. Csuk R. (2019). Hederagenin amide derivatives as potential antiproliferative agents. Eur. J. Med. Chem. 168, 436–446. 10.1016/j.ejmech.2019.02.057 30840925

[B115] Rodríguez-Hernández D. Barbosa L. C. A. Demuner A. J. de Almeida R. M. Fujiwara R. T. Ferreira S. R. (2016a). Highly potent anti-leishmanial derivatives of hederagenin, a triperpenoid from Sapindus saponaria L. Eur. J. Med. Chem. 124, 153–159. 10.1016/j.ejmech.2016.08.030 27569196

[B116] Rodríguez-Hernández D. Barbosa L. C. A. Demuner A. J. Nain-Perez A. Ferreira S. R. Fujiwara R. T. (2017). Leishmanicidal and cytotoxic activity of hederagenin-bistriazolyl derivatives. Eur. J. Med. Chem. 140, 624–635. 10.1016/j.ejmech.2017.09.045 29024910

[B117] Rodríguez-Hernández D. Demuner A. J. Barbosa L. C. Csuk R. Heller L. (2015). Hederagenin as a triterpene template for the development of new antitumor compounds. Eur. J. Med. Chem. 105, 57–62. 10.1016/j.ejmech.2015.10.006 26476750

[B118] Rodríguez-Hernández D. Demuner A. J. Barbosa L. C. Heller L. Csuk R. (2016b). Novel hederagenin-triazolyl derivatives as potential anti-cancer agents. Eur. J. Med. Chem. 115, 257–267. 10.1016/j.ejmech.2016.03.018 27017553

[B119] Ross R. (1993). The pathogenesis of atherosclerosis: a perspective for the 1990s. Nature 362 (6423), 801–809. 10.1038/362801a0 8479518

[B120] Rubinsztein D. C. (2006). The roles of intracellular protein-degradation pathways in neurodegeneration. Nature 443 (7113), 780–786. 10.1038/nature05291 17051204

[B121] Sai Ramesh A. Adarshan S. Lohedan H. Naveen Kumar T. Thasleema Nasrin M. R. Aarthi Shree G. (2023). Computational analysis of the phytocompounds of Mimusops elengi against spike protein of SARS CoV2 - an Insilico model. Int. J. Biol. Macromol. 245, 125553. 10.1016/j.ijbiomac.2023.125553 37356683 PMC10289265

[B122] Saleem S. (2024). Targeting MAPK signaling: a promising approach for treating inflammatory lung disease. Pathol. Res. Pract. 254, 155122. 10.1016/j.prp.2024.155122 38246034

[B123] Selkoe D. J. (2001). Alzheimer's disease: genes, proteins, and therapy. Physiol. Rev. 81 (2), 741–766. 10.1152/physrev.2001.81.2.741 11274343

[B124] Shang Y. Wang Q. Wu B. Zhao Q. Li J. Huang X. (2019). Platelet-membrane-camouflaged black phosphorus quantum dots enhance anticancer effect mediated by apoptosis and autophagy. ACS Appl. Mater Interfaces 11 (31), 28254–28266. 10.1021/acsami.9b04735 31291079

[B125] Sharma D. Khan A. U. (2018). Role of cell division protein divIVA in *Enterococcus faecalis* pathogenesis, biofilm and drug resistance: a future perspective by *in silico* approaches. Microb. Pathog. 125, 361–365. 10.1016/j.micpath.2018.10.001 30290265

[B126] Shen L. Zhang P. Wang J. Ji P. (2020). Tac2-N serves an oncogenic role and promotes drug resistance in human gastric cancer cells. Exp. Ther. Med. 20 (5), 113. 10.3892/etm.2020.9241 32989391 PMC7517536

[B127] Shen Y. Teng L. Qu Y. Huang Y. Peng Y. Tang M. (2023). Hederagenin suppresses inflammation and cartilage degradation to ameliorate the progression of osteoarthritis: an *in vivo* and *in vitro* study. Inflammation 46 (2), 655–678. 10.1007/s10753-022-01763-5 36348189

[B128] Shi R. M. Geng D. S. Li X. X. (2012). Determination of hederagenin and oleanic acid in seed of Nigella glandulifera by HPLC. Pharm. J. Chin. PLA 28 (3), 245–247. 10.3969/j.issn.1008-9926.2012.03.17

[B129] Shi Y. Ma Y. M. Kang Y. X. Wang N. H. (2014). Chemical compositions of Paeonia mairei. Chin. J. Exp. Tradit. Med. Formulae 20 (23), 104–106. 10.13422/j.cnki.syfjx.2014230104

[B130] Shi Z. To S. K. Y. Zhang S. Deng S. Artemenko M. Zhang M. (2021). Hypoxia-induced Nur77 activates PI3K/Akt signaling via suppression of Dicer/let-7i-5p to induce epithelial-to-mesenchymal transition. Theranostics 11 (7), 3376–3391. 10.7150/thno.52190 33537093 PMC7847671

[B131] Shrestha A. Pun N. T. Park P. H. (2018). ZFP36L1 and AUF1 induction contribute to the suppression of inflammatory mediators expression by globular adiponectin via autophagy induction in macrophages. Biomol. Ther. Seoul. 26 (5), 446–457. 10.4062/biomolther.2018.078 30001609 PMC6131013

[B132] Shu K. Cheng T. Chang L. Xu M. (2019). Determination of hederagenin in the bark of Kalopanax septemlobus by HPLC. J Chin Ethnomedicine Ethnopharmacy 28 (12), 39–41.

[B133] Song M. Teng Z. Li M. Niu X. Wang J. Deng X. (2017). Epigallocatechin gallate inhibits Streptococcus pneumoniae virulence by simultaneously targeting pneumolysin and sortase A. J. Cell. Mol. Med. 21 (10), 2586–2598. 10.1111/jcmm.13179 28402019 PMC5618700

[B134] Song M. M. Shang Z. C. Fu X. X. Yu D. Y. Wang Y. Q. Shi L. Y. (2014). Study on the chemical constituents in the stem of Perilla frutescens. China Pharm. 25 (31), 2947–2948. 10.6039/j.issn.1001-0408.2014.31.25

[B135] Sonne D. P. (2021). Mechanisms in Endocrinology: FXR signalling: a novel target in metabolic diseases. Eur. J. Endocrinol. 184 (5), 193–205. 10.1530/eje-20-1410 33630750

[B136] Su F. Sui X. Xu J. Liu Q. Li J. Liu W. (2024). Hederagenin suppresses ovarian cancer via targeting mitochondrial fission through dynamin-related protein 1. Eur. J. Pharmacol. 963, 176188. 10.1016/j.ejphar.2023.176188 37951490

[B137] Subotički T. Mitrović Ajtić O. Beleslin-Čokić B. B. Bjelica S. Djikić D. Diklić M. (2019). IL-6 stimulation of DNA replication is JAK1/2 mediated in cross-talk with hyperactivated ERK1/2 signaling. Cell Biol. Int. 43 (2), 192–206. 10.1002/cbin.11084 30571852 PMC6347554

[B138] Sui X. Chen R. Wang Z. Huang Z. Kong N. Zhang M. (2013). Autophagy and chemotherapy resistance: a promising therapeutic target for cancer treatment. Cell Death Dis. 4 (10), e838. 10.1038/cddis.2013.350 24113172 PMC3824660

[B139] Sung S. A. Kim D. H. Oh K. H. Han S. Y. Han K. H. (2019). The role of cathepsin B in peritoneal fibrosis due to peritoneal dialysis. Int. J. Nephrol. 2019, 4150656. 10.1155/2019/4150656 31815017 PMC6878782

[B140] Susnow N. Zeng L. Margineantu D. Hockenbery D. M. (2009). Bcl-2 family proteins as regulators of oxidative stress. Semin. Cancer Biol. 19 (1), 42–49. 10.1016/j.semcancer.2008.12.002 19138742 PMC4770790

[B141] Takagi K. Park E. H. Kato H. (1980). Anti-inflammatory activities of hederagenin and crude saponin isolated from Sapindus mukorossi Gaertn. Chem. Pharm. Bull. (Tokyo) 28 (4), 1183–1188. 10.1248/cpb.28.1183 7418110

[B142] Tang H. Wang C. Xing C. Liang G. Guo C. Liu X. (2024). Molecular mechanism analysis of the effect of hederagenin combined with L-OHP on chemosensitivity of AGS/L-OHP based on network pharmacology. Curr. Comput. Aided Drug Des. 2024. 10.2174/0115734099270389240104050955 PMC12376131 38243937

[B143] Tian K. Su Y. Ding J. Wang D. Zhan Y. Li Y. (2020). Hederagenin protects mice against ovariectomy-induced bone loss by inhibiting RANKL-induced osteoclastogenesis and bone resorption. Life Sci. 244, 117336. 10.1016/j.lfs.2020.117336 31972206

[B144] Troubat R. Barone P. Leman S. Desmidt T. Cressant A. Atanasova B. (2021). Neuroinflammation and depression: a review. Eur. J. Neurosci. 53 (1), 151–171. 10.1111/ejn.14720 32150310

[B145] Vo N. N. Q. Fukushima E. O. Muranaka T. (2017). Structure and hemolytic activity relationships of triterpenoid saponins and sapogenins. J. Nat. Med. 71 (1), 50–58. 10.1007/s11418-016-1026-9 27491744

[B146] Volkmann N. Marassi F. M. Newmeyer D. D. Hanein D. (2014). The rheostat in the membrane: BCL-2 family proteins and apoptosis. Cell Death Differ. 21 (2), 206–215. 10.1038/cdd.2013.153 24162659 PMC3890954

[B147] Wamil M. Seckl J. R. (2007). Inhibition of 11beta-hydroxysteroid dehydrogenase type 1 as a promising therapeutic target. Drug Discov. Today 12 (13-14), 504–520. 10.1016/j.drudis.2007.06.001 17631244

[B148] Wang B. Liu S. Huang W. Ma M. Chen X. Zeng W. (2021). Design, synthesis, and biological evaluation of hederagenin derivatives with improved aqueous solubility and tumor resistance reversal activity. Eur. J. Med. Chem. 211, 113107. 10.1016/j.ejmech.2020.113107 33360797

[B149] Wang C. Zhou G. L. Vedantam S. Li P. Field J. (2008). Mitochondrial shuttling of CAP1 promotes actin- and cofilin-dependent apoptosis. J. Cell Sci. 121 (Pt 17), 2913–2920. 10.1242/jcs.023911 18716285 PMC2661149

[B150] Wang J. Qin B. H. Yuan Q. Y. Han H. Y. Liu X. Q. (2018a). Chemical constituents of Caulophyllum robustum and its antitumor activities. Chin. Tradit. Herb. Drugs 49 (22), 5242–5246. 10.7501/j.issn.0253-2670.2018.22.004

[B151] Wang K. Liu X. Liu Q. Ho I. H. Wei X. Yin T. (2020). Hederagenin potentiated cisplatin- and paclitaxel-mediated cytotoxicity by impairing autophagy in lung cancer cells. Cell Death Dis. 11 (8), 611. 10.1038/s41419-020-02880-5 32792495 PMC7426971

[B152] Wang L. Zhao M. (2022). Suppression of NOD-like receptor protein 3 inflammasome activation and macrophage M1 polarization by hederagenin contributes to attenuation of sepsis-induced acute lung injury in rats. Bioengineered 13 (3), 7262–7276. 10.1080/21655979.2022.2047406 35266443 PMC9208453

[B153] Wang P. Song Q. S. Xu W. Li S. Z. (2009). Study on the chemical composition of Beaumontia grandiflora branches and leaves. Chin. Tradit. Herb. Drugs 40 (10), 1549–1551.

[B154] Wang X. Liu D. Huang H. Z. Wang Z. H. Hou T. Y. Yang X. (2018b). A novel MicroRNA-124/PTPN1 signal pathway mediates synaptic and memory deficits in Alzheimer's disease. Biol. Psychiatry 83 (5), 395–405. 10.1016/j.biopsych.2017.07.023 28965984

[B155] Wang X. Ren Q. W. Liu X. X. Yang Y. T. Wang B. H. Zhai R. (2019a). Synthesis and biological evaluation of novel H6 analogues as drug resistance reversal agents. Eur. J. Med. Chem. 161, 364–377. 10.1016/j.ejmech.2018.10.033 30384042

[B156] Wang X. Y. Gao H. Zhang W. Li Y. Cheng G. Sun X. L. (2013). Bioactive oleanane-type saponins from the rhizomes of Anemone taipaiensis. Bioorg. Med. Chem. Lett. 23 (20), 5714–5720. 10.1016/j.bmcl.2013.08.006 23992864

[B157] Wang Y. Shou Z. Fan H. Xu M. Chen Q. Tang Q. (2019b). Protective effects of oxymatrine against DSS-induced acute intestinal inflammation in mice via blocking the RhoA/ROCK signaling pathway. Biosci. Rep. 39. 10.1042/bsr20182297 PMC6639456 31262973

[B212] Wen X. Klionsky D. J. (2020). At a glance: a history of autophagy and cancer. Semin. Cancer Biol. 66, 3–11. 10.1016/j.semcancer.2019.11.005 31707087 PMC7202961

[B158] Wong R. S. (2011). Apoptosis in cancer: from pathogenesis to treatment. J. Exp. Clin. Cancer Res. 30 (1), 87. 10.1186/1756-9966-30-87 21943236 PMC3197541

[B159] Wu A. G. Zeng W. Wong V. K. Zhu Y. Z. Lo A. C. Liu L. (2017). Hederagenin and α-hederin promote degradation of proteins in neurodegenerative diseases and improve motor deficits in MPTP-mice. Pharmacol. Res. 115, 25–44. 10.1016/j.phrs.2016.11.002 27838509

[B160] Wu R. Dong S. Cai F. F. Chen X. L. Yang M. D. Liu P. (2019a). Active compounds derived from fuzheng huayu formula protect hepatic parenchymal cells from apoptosis based on network pharmacology and transcriptomic analysis. Molecules 24 (2), 338. 10.3390/molecules24020338 30669350 PMC6358846

[B161] Wu R. Li X. Y. Wang W. H. Cai F. F. Chen X. L. Yang M. D. (2019b). Network pharmacology-based study on the mechanism of bushen-jianpi decoction in liver cancer treatment. Evid. Based Complement. Altern. Med. 2019, 3242989. 10.1155/2019/3242989 PMC6444272 31015849

[B162] Wu S. H. Wu D. G. Chen Y. W. Peng Q. (2005). Chemical constituents of Paeonia delavayi. Chin. Tradit. Herb. Drugs 36 (5), 648–651.

[B163] Xie K. H. Liu X. H. Jia J. Zhong X. Han R. Y. Tan R. Z. (2022a). Hederagenin ameliorates cisplatin-induced acute kidney injury via inhibiting long non-coding RNA A330074k22Rik/Axin2/β-catenin signalling pathway. Int. Immunopharmacol. 112, 109247. 10.1016/j.intimp.2022.109247 36155281

[B164] Xie W. Fang X. Li H. Lu X. Yang D. Han S. (2023a). Advances in the anti-tumor potential of hederagenin and its analogs. Eur. J. Pharmacol. 959, 176073. 10.1016/j.ejphar.2023.176073 37742813

[B165] Xie Z. Wei L. Chen J. Chen Z. (2022b). LncRNA NORAD deficiency alleviates kidney injury in mice and decreases the inflammatory response and apoptosis of lipopolysaccharide-stimulated HK-2 cells via the miR-577/GOLPH3 axis. Cytokine 153, 155844. 10.1016/j.cyto.2022.155844 35255377

[B166] Xie Z. S. Zhao J. P. Wu L. M. Chu S. Cui Z. H. Sun Y. R. (2023b). Hederagenin improves Alzheimer's disease through PPARα/TFEB-mediated autophagy. Phytomedicine 112, 154711. 10.1016/j.phymed.2023.154711 36809694

[B167] Xing L. Chang X. Shen L. Zhang C. Fan Y. Cho C. (2021). Progress in drug delivery system for fibrosis therapy. Asian J. Pharm. Sci. 16 (1), 47–61. 10.1016/j.ajps.2020.06.005 33613729 PMC7878446

[B168] Xu J. Wu K. J. Jia Q. J. Ding X. F. (2020). Roles of miRNA and lncRNA in triple-negative breast cancer. J. Zhejiang Univ. Sci. B 21 (9), 673–689. 10.1631/jzus.B1900709 32893525 PMC7519626

[B169] Xu L. Cui W. H. Zhou W. C. Li D. L. Li L. C. Zhao P. (2017). Activation of Wnt/β-catenin signalling is required for TGF-β/Smad2/3 signalling during myofibroblast proliferation. J. Cell Mol. Med. 21 (8), 1545–1554. 10.1111/jcmm.13085 28244647 PMC5542906

[B170] Xu Q. M. Chen G. Q. Fan J. Y. Zhang M. J. Li X. Yang S. L. (2009). Chemical constituents of roots of Boehmeria nivea. China J. Chin. Mater Med. 34 (20), 2610–2612. 20069903

[B171] Xu S. Y. Xiao Q. W. Zhao S. M. Guan Y. J. Yuan L. H. Zhu Y. (2023). A new cinnamic acid ester derivative from Liquidambaris Resina. China J. Chin. Mater Med. 48 (15), 4130–4136. 10.19540/j.cnki.cjcmm.20230426.201 37802781

[B172] Xue W. Men S. Liu R. (2020). Rotenone restrains the proliferation, motility and epithelial-mesenchymal transition of colon cancer cells and the tumourigenesis in nude mice via PI3K/AKT pathway. Clin. Exp. Pharmacol. Physiol. 47 (8), 1484–1494. 10.1111/1440-1681.13320 32282954 PMC7384028

[B173] Yan H. Li X. Ni W. Zhao Q. Leng Y. Liu H. Y. (2021). Phytochemicals from the leaves of cyclocarya paliurus and their 11β-HSD1 enzyme inhibitory effects. Chem. Biodivers. 18 (1), e2000772. 10.1002/cbdv.202000772 33369207

[B174] Yan Z. Li S. Wang Y. Li J. Ma C. Guo Y. (2022). Discovery of novel heterocyclic derivatives as potential glycogen phosphorylase inhibitors with a cardioprotective effect. Bioorg. Chem. 129, 106120. 10.1016/j.bioorg.2022.106120 36108587

[B175] Yang G. Yang W. Jiang H. Yi Q. Ma W. (2023). Hederagenin inhibits high glucose-induced fibrosis in human renal cells by suppression of NLRP3 inflammasome activation through reducing cathepsin B expression. Chem. Biol. Drug Des. 102, 1409–1420. 10.1111/cbdd.14332 37599208

[B176] Yang H. T. Chen Y. S. Xie J. M. Zhao S. N. (1986). Studies on chemical constituents of Pittosporum brevicalyx (oliv.) gagnep. Acta Chim. Sin. 9, 946–950.

[B177] Yang L. Ritchie A. M. Melton D. W. (2017). Disruption of DNA repair in cancer cells by ubiquitination of a destabilising dimerization domain of nucleotide excision repair protein ERCC1. Oncotarget 8 (33), 55246–55264. 10.18632/oncotarget.19422 28903417 PMC5589656

[B178] Yang M. Wang J. Wang Q. (2022). Hederagenin exerts potential antilipemic effect via p38MAPK pathway in oleic acid-induced HepG2 cells and in hyperlipidemic rats. An. Acad. Bras. Cienc. 94 (4), e20201909. 10.1590/0001-3765202220201909 36102390

[B179] Yang W. He L. (2022). The protective effect of hederagenin on renal fibrosis by targeting muscarinic acetylcholine receptor. Bioengineered 13 (4), 8689–8698. 10.1080/21655979.2022.2054596 35322725 PMC9161953

[B180] Yang X. Li G. Chen L. Zhang C. Wan X. Xu J. (2011a). Quantitative determination of hederagenin in rat plasma and cerebrospinal fluid by ultra fast liquid chromatography-tandem mass spectrometry method. J. Chromatogr. B Anal. Technol. Biomed. Life Sci. 879 (21), 1973–1979. 10.1016/j.jchromb.2011.05.029 21680262

[B181] Yang Y. Guan D. Lei L. Lu J. Liu J. Q. Yang G. (2018). H6, a novel hederagenin derivative, reverses multidrug resistance *in vitro* and *in vivo* . Toxicol. Appl. Pharmacol. 341, 98–105. 10.1016/j.taap.2018.01.015 29408042

[B182] Yang Y. J. Sha C. W. Chen M. G. (2011b). Constituents of Viscum ovalifolium DC.(Ⅱ). Chin. Pharm. J. 46 (1), 11–13.

[B183] Yu H. Song L. Cao X. Li W. Zhao Y. Chen J. (2020). Hederagenin attenuates cerebral ischaemia/reperfusion injury by regulating MLK3 signalling. Front. Pharmacol. 11, 1173. 10.3389/fphar.2020.01173 32848779 PMC7406912

[B184] Yu T. Cheng H. Li X. Huang W. Li H. Gao X. (2023). Design and synthesis of hederagenin derivatives modulating STING/NF-κB signaling for the relief of acute liver injury in septic mice. Eur. J. Med. Chem. 245 (Pt 1), 114911. 10.1016/j.ejmech.2022.114911 36379106

[B185] Zeng J. Huang T. Xue M. Chen J. Feng L. Du R. (2018). Current knowledge and development of hederagenin as a promising medicinal agent: a comprehensive review. RSC Adv. 8 (43), 24188–24202. 10.1039/c8ra03666g 35539158 PMC9082113

[B186] Zhang D. Sun J. Yang B. Ma S. Zhang C. Zhao G. (2020). Therapeutic effect of tetrapanax papyriferus and hederagenin on chronic neuropathic pain of chronic constriction injury of sciatic nerve rats based on KEGG pathway prediction and experimental verification. Evid. Based Complement. Altern. Med. 2020, 2545806. 10.1155/2020/2545806 PMC7306840 32617100

[B187] Zhang H. Jing F. Zhang Z. (2017). Development and validation of a quantification method for oleanolic acid and hederagenin in rat plasma: application to the pharmacokinetic study. Biomed. Chromatogr. 31 (2). 10.1002/bmc.3801 27465077

[B188] Zhang L. J. Cheng J. J. Liao C. C. Cheng H. L. Huang H. T. Kuo L. M. (2012). Triterpene acids from Euscaphis japonica and assessment of their cytotoxic and anti-NO activities. Planta Med. 78 (14), 1584–1590. 10.1055/s-0032-1315040 22814889

[B189] Zhang R. Zhu H. Ding L. Yang Z. (2014). Determination of asperosaponin VI and its active metabolite hederagenin in rat tissues by LC-MS/MS: application to a tissue distribution study. J. Chromatogr. B Anal. Technol. Biomed. Life Sci. 959, 22–26. 10.1016/j.jchromb.2014.03.030 24747520

[B190] Zhang R. H. Jin R. Deng H. Shen Q. K. Quan Z. S. Jin C. M. (2021a). Evaluation of the anti-toxoplasma gondii activity of hederagenin *in vitro* and *in vivo* . Korean J. Parasitol. 59 (3), 297–301. 10.3347/kjp.2021.59.3.297 34218602 PMC8255499

[B191] Zhang W. Lv M. Shi Y. Mu Y. Yao Z. Yang Z. (2021b). Network pharmacology-based study of the underlying mechanisms of huangqi sijunzi decoction for Alzheimer's disease. Evid. Based Complement. Altern. Med. 2021, 6480381. 10.1155/2021/6480381 PMC8510793 34650613

[B192] Zhang W. Xu C. Sun J. Shen H. M. Wang J. Yang C. (2022a). Impairment of the autophagy-lysosomal pathway in Alzheimer's diseases: pathogenic mechanisms and therapeutic potential. Acta Pharm. Sin. B 12 (3), 1019–1040. 10.1016/j.apsb.2022.01.008 35530153 PMC9069408

[B193] Zhang X. He B. H. Yang X. Li L. Y. (2008). Determination of Hederagenin, oleanolic acid, quercetin, lignan and chlorogenic acid in *Lonicera japonica* Thunb by HPLC. Chin. Tradit. Herb. Drugs 39 (10).

[B194] Zhang Y. Feng W. Peng X. Zhu L. Wang Z. Shen H. (2022b). Parthenolide alleviates peritoneal fibrosis by inhibiting inflammation via the NF-κB/TGF-β/Smad signaling axis. Lab. Invest. 102 (12), 1346–1354. 10.1038/s41374-022-00834-3 36307537

[B195] Zhang Y. Han Y. Shang Y. Wang X. Sun J. (2023). Proteomics identifies differentially expressed proteins in glioblastoma U87 cells treated with hederagenin. Proteome Sci. 21 (1), 7. 10.1186/s12953-023-00208-7 37120556 PMC10148390

[B196] Zhang Y. H. Wang T. Lu Z. G. Wang H. Q. (2002). Studies on chemical constituents of Galeopsis bifida. China J. Chin. Mater Med. 27 (3), 206–208. 12774403

[B197] Zhang Z. L. Zuo Y. M. Luo G. M. Fu X. M. Cai C. J. Wang Y. Y. (2013). Studies on the chemical components of triterpenoids of Gardenia jasminoides Ellis. Lishizhen Med. Mater Med. Res. 24 (2), 338–339. 10.3969/j.issn.1008-0805.2013.02.039

[B198] Zhao C. Denison C. Huibregtse J. M. Gygi S. Krug R. M. (2005). Human ISG15 conjugation targets both IFN-induced and constitutively expressed proteins functioning in diverse cellular pathways. Proc. Natl. Acad. Sci. U. S. A. 102 (29), 10200–10205. 10.1073/pnas.0504754102 16009940 PMC1177427

[B199] Zhao L. Chen T. Hang P. Li W. Guo J. Pan Y. (2019). Choline attenuates cardiac fibrosis by inhibiting p38MAPK signaling possibly by acting on M(3) muscarinic acetylcholine receptor. Front. Pharmacol. 10, 1386. 10.3389/fphar.2019.01386 31849653 PMC6900736

[B200] Zhao L. Shi H. Zhang F. Xue H. Han Q. (2023). Hederagenin protects against myocardial ischemia-reperfusion injury via attenuating ALOX5-mediated ferroptosis. Naunyn Schmiedeb. Arch. Pharmacol. 397, 3411–3424. 10.1007/s00210-023-02829-3 37955689

[B201] Zhao Y. X. Liang W. J. Fan H. J. Ma Q. Y. Tian W. X. Dai H. F. (2011). Fatty acid synthase inhibitors from the hulls of Nephelium lappaceum L. Carbohydr. Res. 346 (11), 1302–1306. 10.1016/j.carres.2011.04.028 21605850

[B202] Zheng G. H. Pei X. L. Jin Y. Lv H. Z. (2012). Determination of content of hederagenin in the Pulsatilla dahurica (Fisch.) spreng. J. Med. Sci. Yanbian Univ. 35 (1), 30–32.

[B203] Zheng S. Z. Sun P. Wang J. P. Liu Y. Gong W. Liu J. (2019). MiR-34a overexpression enhances the inhibitory effect of doxorubicin on HepG2 cells. World J. Gastroenterol. 25 (22), 2752–2762. 10.3748/wjg.v25.i22.2752 31235998 PMC6580351

[B204] Zheng Y. Yan L. Y. Chen Y. Xu X. B. (2022). Effects of hederagenin on the biological behavior of TPC-1 thyroid cancer cells via PCAT19/miR-4319 regulation. Chin. Tradit. Pat. Med. 44 (7), 2132–2137. 10.3969/j.issn.1001-1528.2022.07.011

[B205] Zhong S. H. Wei Y. F. Gu R. Wu Y. Duan X. (2010). HPLC determination of hederagenin in leaves of Acanthopanax giraldii Harms. Lishizhen Med. Mater Med. Res. 21 (1), 6–7.

[B206] Zhou D. Jin H. Lin H. B. Yang X. M. Cheng Y. F. Deng F. J. (2010). Antidepressant effect of the extracts from Fructus Akebiae. Pharmacol. Biochem. Behav. 94 (3), 488–495. 10.1016/j.pbb.2009.11.003 19931301

[B207] Zhou X. J. Li Z. L. Li J. Zhou J. Cheng M. J. Qian S. H. (2019). Chemical constituents from Clematis apiifolia. Chin. Tradit. Herb. Drugs 50 (3), 557–562. 10.7501/j.issn.0253-2670.2019.03.004

[B208] Zhou Y. Wang Y. Li Q. Dong K. Chen C. Mao E. (2022). Downregulation of lncRNA NEAT1 alleviates sepsis-induced acute kidney injury. Cent. Eur. J. Immunol. 47 (1), 8–19. 10.5114/ceji.2022.115628 35600150 PMC9115601

[B209] Zhu H. Ding L. Shakya S. Qi X. Hu L. Yang X. (2011). Simultaneous determination of asperosaponin VI and its active metabolite hederagenin in rat plasma by liquid chromatography-tandem mass spectrometry with positive/negative ion-switching electrospray ionization and its application in pharmacokinetic study. J. Chromatogr. B Anal. Technol. Biomed. Life Sci. 879 (30), 3407–3414. 10.1016/j.jchromb.2011.09.014 21963276

[B210] Zhu J. Thompson C. B. (2019). Metabolic regulation of cell growth and proliferation. Nat. Rev. Mol. Cell Biol. 20 (7), 436–450. 10.1038/s41580-019-0123-5 30976106 PMC6592760

